# Structural Basis of Lymphangiogenic Receptor VEGFR‐3 Activation Mediated by Distinctive Clustering of the Ligand–Receptor Complex

**DOI:** 10.1002/advs.77728

**Published:** 2026-09-09

**Authors:** Ryeongeun Cho, Jinsook Ahn, Jimin Yang, Dong Sun Lee, Gahi Hong, Sangkyu Lee, Ho Min Kim

**Affiliations:** ^1^ InnoCORE AI‐CRED Institute Korea Advanced Institute of Science and Technology (KAIST) Daejeon Republic of Korea; ^2^ Graduate School of Medical Science and Engineering Korea Advanced Institute of Science and Technology (KAIST) Daejeon Republic of Korea; ^3^ Department of Biological Sciences Korea Advanced Institute of Science and Technology (KAIST) Daejeon Republic of Korea; ^4^ Department of Molecular Biology Jeonbuk National University Jeonju Republic of Korea; ^5^ Center for Memory and Glioscience Institute for Basic Science (IBS) Daejeon Republic of Korea; ^6^ Research Institute for Materials and Energy Sciences Jeonbuk National University Jeonju Republic of Korea

**Keywords:** biology, cell biology, lymphangiogenesis, structural biology, transmission electron microscopy, vascular endothelial growth factor c, vascular endothelial growth factor receptor

## Abstract

Vascular endothelial growth factor‐C (VEGF‐C) and its receptor VEGFR‐3 are critical for lymphangiogenesis, yet the structural mechanisms beyond simple dimerization that drive activation remain unknown. Here, we report the first cryo‐EM structure of the human VEGFR‐3 full ectodomain in complex with VEGF‐C. Such a canonical ligand‐induced 2:2 hetero‐tetrameric complex further self‐assembles into distinct higher‐order assemblies—a lateral *cis*‐cluster and an inverted *trans*‐like‐cluster. Further analysis of these canonical 2:2 hetero‐tetramer in higher‐order assemblies identifies that the specificity of VEGF‐C for VEGFR‐3 and VEGFR‐2 over VEGFR‐1 is mainly governed by its N‐terminal α1 helix, which is structurally accommodated by the D2 domains of VEGFR‐3 and VEGFR‐2 but sterically excluded by the protruding D1–D2 connecting loop of VEGFR‐1. Furthermore, we identify a unique interface within the membrane‐proximal D5 domain occurring between neighboring canonical 2:2 complexes, mediated by a specific “WTP motif,” as a key driver of *cis*‐clustering. Structure‐guided mutagenesis and real‐time optogenetic assays support a critical role for *cis*‐clustering in signal amplification and show that enforced receptor clustering can drive robust activation, whereas *trans*‐like‐clustering appears dispensable. Collectively, these findings transform the traditional “monomer‐to‐dimer” activation model of VEGFR‐3/VEGF‐C into a “dimer‐to‐cluster” paradigm, providing a blueprint for engineering next‐generation therapeutics targeting lymphatic vascular diseases.

## Graphical abstract

VEGF‐C and its receptor VEGFR‐3 are critical for lymphangiogenesis. Cryo‐EM structures of the VEGFR‐3/VEGF‐C ectodomain complex reveal a canonical 2:2 ligand–receptor hetero‐tetramer that further assembles into higher‐order clusters beyond simple receptor dimerization. Structure‐guided mutagenesis and optogenetic clustering show that *cis*‐clustering of VEGFR‐3/VEGF‐C complex is essential for signal amplification, establishing a dimer‐to‐cluster paradigm for lymphangiogenic VEGFR‐3 signaling.

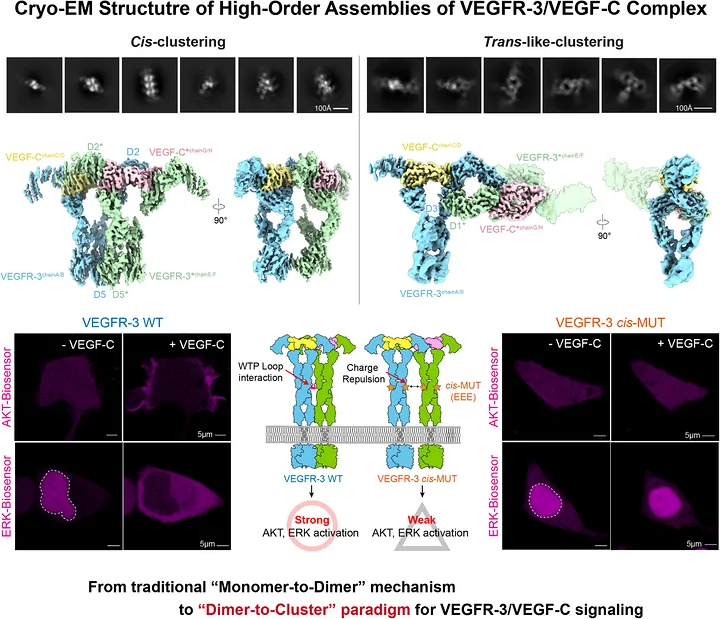

## 1 Introduction

The vascular system, comprising interconnected blood and lymphatic vessels, is essential for maintaining tissue homeostasis, fluid balance, and immune surveillance. Vascular endothelial growth factors (VEGFs; VEGF‐A, VEGF‐B, VEGF‐C, VEGF‐D, and PlGF) enable signal transmission through three cognate receptors—VEGFR‐1, ‐2, and ‐3—each playing distinct roles in vasculogenesis, angiogenesis, and lymphangiogenesis. VEGF‐A signaling is mediated predominantly via VEGFR‐2 and drives the angiogenic sprouting of blood vessels, whereas the VEGFR‐3/VEGF‐C pathway plays an indispensable role in developmental lymphangiogenesis and maintaining lymphatic function [1, 2]. Therefore, a dysregulation of this VEGFR‐3/VEGF‐C pathway has severe consequences: loss‐of‐function mutations cause hereditary lymphedema (e.g., Milroy's disease), whereas an enhancement in the associated signaling promotes tumor‐associated lymphangiogenesis, facilitating cancer metastasis [3, 4, 5]; thus, it represents a compelling therapeutic target. Enhancing such signaling (e.g., with recombinant VEGF‐C protein‐ or mRNA‐based therapy) may ameliorate lymphedema [6, 7, 8], whereas its suppression can restrain tumor lymphangiogenesis and metastasis with approaches ranging from ligand traps to receptor‐blocking antibodies or small molecules still under investigation [2, 3, 4, 5]. These physiological and clinical insights highlight the significance of understanding the VEGFR‐3/VEGF‐C signaling mechanism at the molecular level.

VEGFRs belong to the type‐V subfamily of receptor tyrosine kinases (RTKs) and share a common modular architecture consisting of seven extracellular immunoglobulin (Ig)‐like domains (D1–D7), a single‐pass transmembrane helix, and an intracellular tyrosine kinase domain [9]. All VEGF family ligands are secreted as propeptides and proteolytically processed to generate bioactive homodimers [10]. These active ligands form antiparallel cystine‐knot dimers covalently linked by two intermolecular disulfide bonds and, as bivalents, bridge two receptor monomers through contacts mainly present in the D2–D3 domain. The D2 domain common to VEGFRs is the principal determinant of high‐affinity ligand binding, with the D3 domain stabilizing the ligand–receptor complex by providing additional points of contact [11, 12]. Concomitantly, the membrane‐proximal regions, D4–D7, form homotypic interactions that further stabilize this dimer [13, 14]. Such ligand‐induced dimerization rigidly aligns the two receptor chains, thereby juxtaposing their intracellular kinase domains and triggering autophosphorylation as well as downstream signaling [15]. This canonical mechanism of VEGFR activation is analogous to other RTKs and has been supported by biochemical and structural studies on members of the VEGFR family. However, emerging evidence from reports on other RTKs, such as EGFR and Eph receptors, suggests that the signaling output is not merely governed by simple dimerization but is often amplified or regulated by the formation of higher‐order oligomers or clusters on the cell membrane [16, 17, 18, 19, 20], while little is known regarding VEGF/VEGFR clustering.

Despite this overall paradigm, VEGFR‐3 exhibits unique structural features. Unlike VEGFR‐1 or ‐2, it matures via proteolytic cleavage within an extended loop of D5 after receptor biosynthesis [21, 22]. Moreover, disulfide bonds within D5 stabilize this region and preserve receptor integrity despite proteolytic cleavage. Although the cleavage itself is not strictly required for receptor activity, the loop structure and its internal disulfide bonds are crucial for maintaining a D5 conformation that supports receptor function [23]. Consistent with this finding, the anti‐VEGFR‐3 antibody 2E11, which recognizes a conformational epitope present within this D5 loop, inhibits ligand‐induced signal transduction as well as the migration and sprouting of microvascular endothelial cells [23].

While the full‐ectodomain (ECD) structure of VEGFR‐1 in complex with VEGF‐A has been elucidated by X‐ray crystallography [13], the complete architecture of the VEGFR‐3 ectodomain in complex with its ligand VEGF‐C has remained elusive. Previous structural studies have been limited to partial complexes, such as the VEGFR‐3 D4–D5 homodimer and VEGF‐C bound to the VEGFR‐3 D1–D2 or VEGFR‐2 D2–D3 domains [14, 24]. Moreover, the partially available VEGFR‐3 D1–D2/VEGF‐C structure, determined at a moderate resolution of ∼4.6 Å, is insufficient to define in detail the intermolecular interaction. Additionally, the mechanism by which the binding of VEGF‐C at the distal domains is mechanically transmitted through the entire VEGFR‐3 extracellular region to coordinate the precise assembly required for robust signal transduction remains unclear.

Here, we present the first structure of the entire human VEGFR‐3 ECD in complex with VEGF‐C. Using single‐particle cryo‐electron microscopy (cryo‐EM), we show that VEGF‐C induces the canonical 2:2 VEGFR‐3/VEGF‐C complex, which can further self‐assemble into two distinct higher‐order oligomer arrangements. Structure‐guided mutagenesis reveals that one of these assembly modes—a lateral *cis*‐cluster mediated by a unique interface within the VEGFR‐3 D5 domain—is crucial for downstream signaling, whereas the alternative *trans*‐like‐cluster interface is dispensable. Furthermore, by leveraging an optogenetic clustering system, we demonstrate that the enforced oligomerization of VEGFR‐3 is sufficient to drive robust signaling in the absence of any ligand. Together, our findings unveil an unanticipated structural plasticity of the VEGFR‐3/VEGF‐C complex and establish higher‐order receptor clustering as a key mechanism for amplifying VEGFR‐3–mediated lymphatic signaling.

## 2 Results

### 2.1 Overall Structure of the Higher‐Order Assemblies of the Human VEGFR‐3/VEGF‐C Complex

**FIGURE 1:**
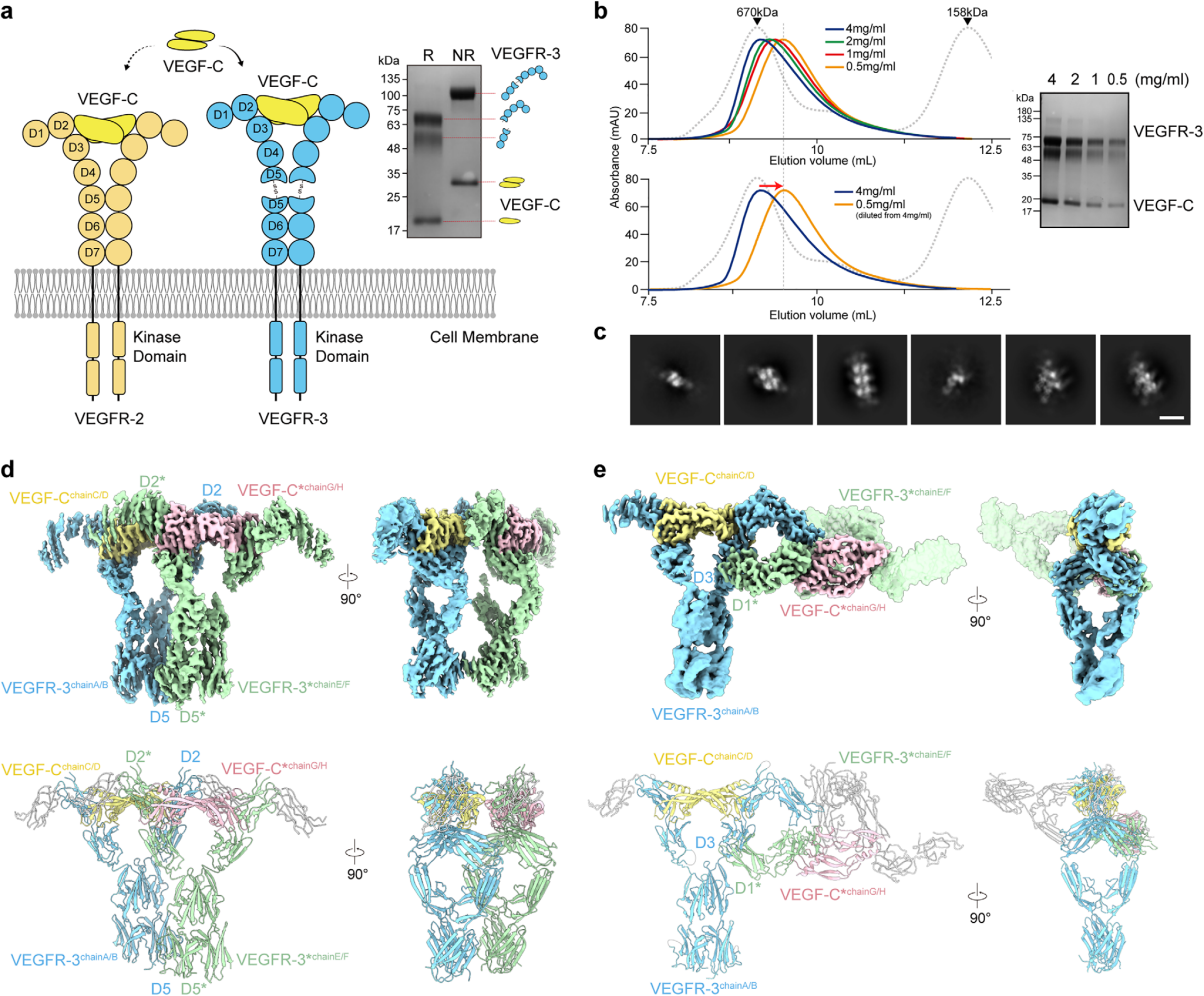
Overall structure of the higher‐order assemblies of human VEGFR‐3/VEGF‐C complex. a Domain architecture of the human VEGF‐C and its receptor interactions, VEGFR‐2, and VEGFR‐3. VEGFR‐3 undergoes proteolytic cleavage within the D5 domain after receptor biosynthesis, but the resulting fragments remain linked via a disulfide bridge. Reducing (R) and non‐reducing (NR) SDS‐PAGE analysis of the purified VEGFR‐3 ECD/VEGF‐C complex (right). b Concentration‐dependent oligomerization of the VEGFR‐3 ECD/VEGF‐C complex analyzed by size‐exclusion chromatography (SEC). The elution peak shift upon dilution from 4 to 0.5 mg/ml is displayed in the lower panel. Grey dashed lines indicate the elution volumes of the molecular weight standards. Reducing SDS‐PAGE analysis of the highest‐peak fractions of SEC with the concentrations indicated (right). c Representative 2D class averages of the VEGFR‐3 ECD/VEGF‐C complexes with the 2 mg/ml SEC fraction (green profile in b). Scale bar: 100 Å. d and e Cryo‐EM density maps (top) and corresponding atomic models (bottom) of the *cis*‐clustered VEGFR‐3 ECD/VEGF‐C complex (d) and the *trans*‐like‐clustered VEGFR‐3 ECD/VEGF‐C complex (e), shown in two orthogonal views. A single complex unit is colored (VEGFR‐3, blue and dimeric VEGF‐C, yellow), with the adjacent interacting unit colored light green (VEGFR‐3*) and pink (VEGF‐C*). An unresolved domain is indicated with a gray ribbon. Regions of the adjacent VEGFR‐3* with unresolved densities are rendered as transparent green surfaces (e).

To elucidate the atomic details of the VEGFR‐3/VEGF‐C complex, we first co‐expressed the human VEGFR‐3 ECD (D1–D7) and a mature form of VEGF‐C (residues 103–215, C137A) [25] in Expi293F cells (Figure 1a). The complex was purified by protein A affinity chromatography, Fc tag removal via on‐resin 3C protease cleavage, and subsequent size‐exclusion chromatography (SEC). Reducing and non‐reducing SDS‐PAGE analyses confirmed that the mature VEGF‐C formed dimers through disulfide linkages [26], and that the proteolytically cleaved VEGFR‐3 ECD was correctly assembled via disulfide bridges [22] (Figure 1a).

Consistent with the findings of previous studies [14, 27], negative‐staining EM analysis of the purified VEGFR‐3 ECD/VEGF‐C complex at a low concentration of 14.5 µg/ml revealed discrete, Y‐shaped particles corresponding to the canonical 2:2 hetero‐tetrameric complex, hereafter, indicated as [VEGFR‐3/VEGF‐C]_2_, despite the membrane‐proximal region being resolved only partially (Figure S1a). Surprisingly, when the complex was concentrated to 1 mg/ml for cryo‐EM, raw micrographs showed elongated and filament‐like structures rather than the monodispersed particles of the canonical [VEGFR‐3/VEGF‐C]_2_ complex (Figures S1b,c). Interestingly, 2D class averages revealed two distinct oligomerization modes: 1) straight, short filaments with regularly spaced lattice‐like arrays, or 2) more irregular, longer, and slightly bent arrangements.

To ensure that these oligomeric assemblies were not non‐specific aggregation artifacts, we employed size‐exclusion chromatography (SEC) to analyze their concentration‐dependent oligomerization status across a range of 0.5 to 4.0 mg/ml. The SEC profiles demonstrated a progressive peak shift toward higher molecular weight species with increasing protein concentration (Figure 1b, top), confirming a dynamic, concentration‐dependent self‐assembly. Even at 0.5 mg/ml, the VEGFR‐3/VEGF‐C complex eluted markedly earlier than the expected ∼300 kDa size of the canonical [VEGFR‐3/VEGF‐C]_2_ complex. The 2D class averages from the peak of the 2 mg/ml SEC fraction of VEGFR‐3/VEGF‐C complex further confirmed these oligomeric assemblies (Figure 1c). These results suggest that the higher‐order, filament‐like structures represent relevant oligomers rather than random aggregates. Importantly, when the high‐concentration samples were diluted from 4 to 0.5 mg/ml, the position of the SEC peak reverted to that of the 0.5 mg/ml profile, indicating that the oligomerization behavior is reversible (Figure 1b, bottom).

To gain structural insights into these higher‐order assemblies, we performed single‐particle cryo‐EM analysis with the purified VEGFR‐3 ECD/VEGF‐C complex at a high concentration of 1 mg/ml. Extensive 2D and 3D classifications identified two distinct oligomeric architectures, each composed of the [VEGFR‐3/VEGF‐C]_2_ unit assembled via specific intermolecular clustering interfaces (Figure 1d,e and Table S1 and Figures S2–S4). The first configuration, which we term the *cis*‐clustered complex, revealed a lateral, “side‐by‐side” alignment of [VEGFR‐3/VEGF‐C]_2_ unit into a continuous lattice‐like array (Figure 1d). Cryo‐EM reconstruction of this assembly achieved an overall resolution of ∼3.3 Å (Figure S2), enabling reliable model building into the cryo‐EM density map, except for the flexible D1 and D6–D7 domains. However, at lower contour thresholds, we observed a globular density putatively assigned to D6, which aligns well with the position of the D6 domain within the VEGFR‐1 D1–D6/VEGF‐A complex.

In contrast, the second form, termed the *trans*‐like‐clustered complex, exhibited an inverted, alternating arrangement between neighboring [VEGFR‐3/VEGF‐C]_2_ complexes and was resolved to ∼3.2 Å (Figure 1e). The core regions—specifically the binding site of dimeric VEGF‐C to VEGFR‐3 D2‐D3 and the reciprocal *trans*‐like‐clustering interface between D1 and D3 of adjacent receptor—were well‐resolved. However, the peripheral regions of the neighboring complexes, particularly, the D4–D7 domains of VEGFR‐3*^chainE^ and the entire VEGFR‐3*^chainF^, remained flexible and undefined (Figure 1e; Table S1, and Figures S3–S4). These two distinct assembly modes suggest that the VEGFR‐3/VEGF‐C complex possesses intrinsic structural plasticity, most likely facilitating the formation of dynamic higher‐order clusters on the lymphatic endothelial cell membrane during signaling.

### 2.2 Architecture and Conformational Plasticity of the Dimeric VEGFR‐3/VEGF‐C Complex

**FIGURE 2:**
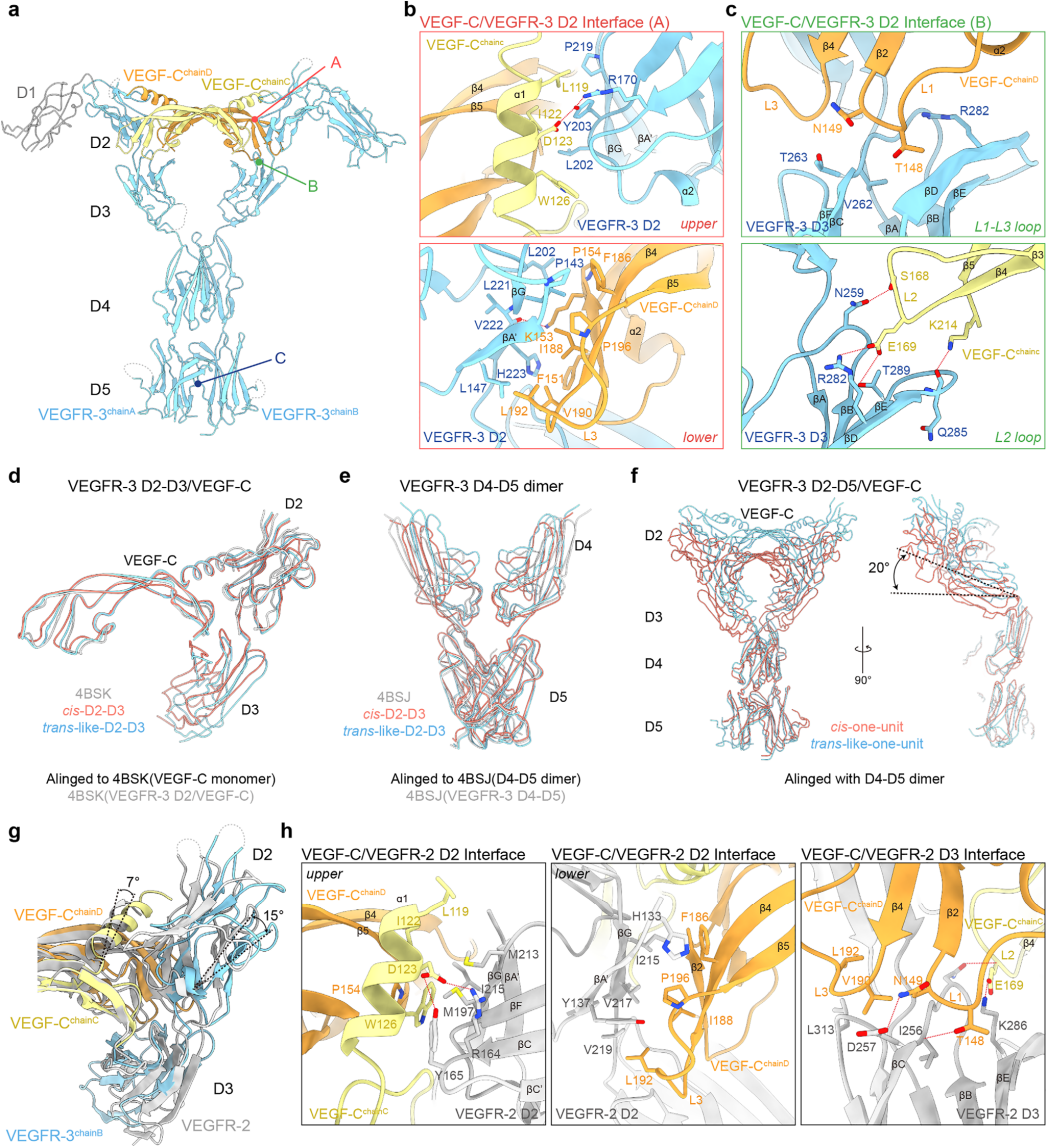
Structural analysis of the dimeric VEGFR‐3 ECD/VEGF‐C complex and its interaction interfaces. a Overall structure of the dimeric VEGFR‐3 ECD/VEGF‐C complex (from a *trans*‐like‐clustered assembly). Key interacting surfaces for VEGF‐C (yellow and orange)‐mediated VEGFR‐3 (cyan) dimerization are highlighted and categorized into three regions: Region A, B, and C. An unresolved D1 domain of VEGFR‐3^chainA^ is indicated with a gray ribbon. b and c Close‐up views of the three interaction regions in VEGFR‐3 and VEGF‐C interface. The residues involved in the VEGFR‐3^chainB^ D2 and VEGF‐C interaction (red box) (b) and the VEGFR‐3^chainB^ D3 and VEGF‐C interaction (green box) (c) are displayed as sticks and labeled. Hydrogen bonds and electrostatic interactions are indicated by red dashed lines. Secondary‐structure elements of VEGF‐C and VEGFR‐3 are also labeled. The three exposed loops of VEGF‐C are designated L1, L2, and L3. d–f Structural comparison of the partial and full VEGFR‐3 D2–D5/VEGF‐C complexes from *cis‐* and *trans‐*like‐clustered assemblies with the previously reported structures (gray). All structures are represented as ribbons. Superimposition of the VEGFR‐3 D1–D2/VEGF‐C protomers from *cis*‐ and *trans*‐like‐clustered assemblies with VEGFR‐3 D2/VEGF‐C (PDB: 4BSK) (d), VEGFR‐3 D4–D5 dimers from cis‐ and *trans*‐like‐clustered assemblies with the D4–D5 dimer (PDB: 4BSJ) (e), [VEGFR‐3 D2–D5/VEGF‐C]_2_ complex from the *cis*‐ with that from *trans*‐like‐cluster assemblies (f). An ∼20° angle between the *cis‐*one‐unit and *trans‐*like‐one‐unit is indicated, with the D3–D4 loop acting as a hinge. g Structural comparison of the VEGFR‐3 D2‐D3/VEGF‐C (cyan) and VEGFR‐2 D2‐3/VEGF‐C (gray, PDB: 2X1W), aligned with the VEGF‐C^chainC^ (Cα atoms RMSD for VEGF‐C^chainC^: 0.630 Å). The angular differences between the corresponding domains, or the N‐terminal α1 helixes were measured and are indicated. h Close‐up views of the VEGFR‐2 and VEGF‐C interfaces. The VEGFR‐2 D2–(left, middle) and D3–mediated (right) contacts with VEGF‐C are highlighted, with secondary‐structure elements labeled.

The fundamental unit of the *cis‐* and *trans*‐like‐clustered assemblies is a 2:2 heterotetramer, in which a dimeric VEGF‐C symmetrically bridges two VEGFR‐3 ECD protomers (Figure 1d,e), resembling the conserved architecture of other complexes belonging to the VEGF/VEGFR family (Figure S5a) [28, 29]. Despite differences in oligomer arrangement, the VEGFR‐3 D2–D3/VEGF‐C protomers in both assemblies are structurally highly similar, indicating a conserved mode of ligand engagement (Figure S5b‐c). Therefore, we leveraged the higher local resolution of the core regions within the *trans*‐like‐clustered complex to delineate the atomic details of the VEGF‐C dimer and ligand–receptor interfaces (Figure 2a; Figure S4c‐e).

Consistent with previous studies [24], the mature VEGF‐C adopted a canonical cystine‐knot motif, forming an antiparallel dimer stabilized by two intermolecular disulfide bonds: Cys156^chainC^–Cys165^chainD^ and Cys156^chainD^–Cys165^chainC^ (Figure 2a; Figures S4c and S6). Our resolved structure shows that the extracellular region of VEGFR‐3, consisting of D1–D5 (residues 26–554), comprises Ig‐like domains, each adopting a β‐sandwich fold. VEGF‐C engages VEGFR‐3 primarily through the D2 and D3 domains, burying ∼706 Å^2^ (57%, Region A) and 527 Å^2^ (43%, Region B) of surface area, respectively (Figure 2a–c; Figure S5d,e). Notably, although the previously reported crystal structure (PDB ID: 4BSK) [14] was limited to D2 and determined at lower resolution, its overall secondary structure is nearly identical to our atomic configuration—Cα atoms RMSD: 1.699 and 1.866 Å for a protomer of the *trans*‐like and *cis*‐clustered assemblies vs. 4BSK, respectively (Figure 2d).

Region A is formed by interactions of VEGFR‐3 D2 with the α1 helix of VEGF‐C^chainC^ (upper), and the β2, β4 and L3 loop of VEGF‐C^chainD^(lower). In the upper part of Region A, a compact hydrophobic core is formed by the VEGFR‐3 D2 residues—L202 and Y203 on the α‐βF loop, and P219 on the βF‐βG loops—which anchor the α1 helix of the VEGF‐C^chainC^ via residues L119, I122, and W126 (Figure 2b, top; Table S2). Such an interaction of the hydrophobic cores is further stabilized by electrostatic bonds between R170–Y203 of VEGFR‐3 D2 and D123 of VEGF‐C^chainC^ (Figure 2b, top; Table S2). The essential role of D123 in receptor binding was confirmed by a previous mutagenesis study [14]. In the lower part of Region A, the hydrophobic residues of VEGFR‐3 D2—P143, L147, L221, and H223—extend the interface, packing against the β2 strand via residues F151, K153, and P154 with the β4 strand–L3 (β4‐β5 loop) of VEGF‐C^chainD^ via the residues F186, I188, V190, L192, and P196 (Figure 2b, bottom Table S2).

In contrast, Region B is primarily mediated by electrostatic interactions between VEGFR‐3 D3 and VEGF‐C (Figure 2c; Table S2). The residues T148 and N149 on the L1 of VEGF‐C^chainD^ (α2‐β2 loop) interact with the main chain of V262 and T263 of VEGFR‐3, respectively (Figure 2c, top and Table S2). More crucially, S168 and E169 on the L2 of VEGF‐C^chainC^ (β3‐β4 loop), along with K214, which are conserved across VEGF‐C orthologs (human, mouse, and rat), engage N259, R282, T289, and the main chain of Q285 on VEGFR‐3 D3 through hydrogen and ionic bonds (Figure 2c, bottom and Table S2). Among these, E169 on L2—and its corresponding residues in other VEGF family members—plays a central role in VEGFR D3 interactions [13, 14, 24]. Notably, although the VEGF‐A/VEGFR‐1 complex engages VEGFR‐1 D3 via the L1, L2, and L3 of VEGF‐A [13], our structure reveals that VEGF‐C interacts with VEGFR‐3 D3 exclusively through L1 and L2, with L3 contributing instead to the engagement with VEGFR‐3 D2.

Region C is characterized by receptor–receptor homodimerization mediated by the D5 domains. Although the local resolution of this membrane‐proximal region was lower than that of the ligand‐binding interface, the D4–D5 arrangement resembles the high‐resolution structure of the isolated D4–D5 homodimer crystal (PDB: 4BSJ) [14], exhibiting only minor angular deviations (Figure 2e). Such structural consistency suggests that the symmetric interface is stabilized by ionic and polar interactions common to the crystal structure, specifically involving K516, H425, T446, and E509 of one protomer with E428 and S430 of the adjacent protomer (Table S2).

Interestingly, a structural comparison of the dimeric VEGFR‐3 D1–D5/VEGF‐C complex in *cis‐* and *trans*‐like‐clustered assemblies revealed that the individual N‐terminal ligand‐binding modules (VEGFR‐3 D1–D3/VEGF‐C) and C‐terminal dimerization modules (VEGFR‐3 D4–D5) align closely; however, the overall architectures differed significantly (RMSD: 19.58 Å) (Figure 2f). Indeed, when the D4–D5 homodimers are superimposed, the upper ligand‐binding module (VEGFR‐3 D1–D3/VEGF‐C) of the *cis*‐clustered assembly is rotated by ∼20° relative to that of the *trans*‐like‐clustered one (Figure 2f, right). Such deviation primarily arises from the flexible linker between D3 and D4, indicating that this hinge‐like motion most likely facilitates the conformational plasticity and structural adaptability required for distinct clusterings.

### 2.3 Structural Comparison of the Modes of VEGF‐C Binding to VEGFR‐2 and VEGFR‐3

To elucidate the structural basis for the modest differences in VEGF‐C binding affinity between VEGFR‐3 (K_D_ = 135 pM) and VEGFR‐2 (K_D_ = 410 pM) [30], we aligned our structure with the previously reported VEGFR‐2 D2–D3/VEGF‐C complex (PDB: 2X1W) [24]. Superposition of the VEGF‐C between the two complexes revealed that the ligand conformation is highly conserved (Cα atoms RMSD: 1.04 Å for the VEGF‐C dimer), with a notable exception of the α1 helix of VEGF‐C^chainC^. In contrast, the D2–D3 domains of the receptor exhibited a substantially larger structural deviation (Cα atoms RMSD: 3.06 Å for D2–D3 domains of VEGFR‐3 vs. VEGFR‐2) (Figure 2g). These differences primarily arise from the distinct orientations of these domains relative to the VEGF‐C dimer. Specifically, the D2 domain of VEGFR‐2 engages VEGF‐C at a steeper angle, ∼15° more upright, compared to that of VEGFR‐3, resulting in a rotation of the key N‐terminal α1 helix within the VEGFR‐2/VEGF‐C complex by ∼7°.

This conformational shift is driven by specific steric constraints. In the VEGFR‐2 D2 domain, the protruding hydrophobic residues M197 and M213 push away the N‐terminal α1 helix of the VEGF‐C, while the residues R164 and Y165 of the hairpin turn βC–βC′ anchor D123 of the VEGF‐C α1 helix, acting as the hinge point (Figure 2h). Furthermore, the interaction topology varied significantly: VEGF‐C engages the surface of the VEGFR‐2 β‐sheet, whereas it interacts with the loops at the edge of the VEGFR‐3 β‐sheet. These geometrical deviations result in a less compact packing of the VEGF‐C α1 helix against the VEGFR‐2 D2 domain. Indeed, the interaction surface within the VEGFR‐3 D2/VEGF‐C complex is slightly larger in area than that in the VEGFR‐2 D2/VEGF‐C complex (Table S3), providing a plausible structural basis that may contribute to the slightly lower affinity of VEGF‐C to VEGFR‐2.

Similar to D2 domains, the D3 also exhibit slight structural deviations between the VEGFR‐2 and VEGFR‐3 complexes (Figure 2g). However, our analysis of the seven VEGFR‐3 protomers across *cis‐* and *trans*‐like‐assemblies reveals that the relative positions of D2 and D3 among them are nearly identical (Figure S5b‐c). This observation contrasts with previous structural studies showing that the relative orientation of D2 and D3 in VEGFR‐2 is inherently plastic, varying remarkably across different crystalline forms and protomers [24]. These findings suggest that the VEGF‐C/VEGFR‐3 D3 interface is more tightly constrained than that within VEGF‐C/VEGFR‐2 D3.

### 2.4 Structural Determinants of the *cis‐* and *trans‐*like‐Clustering of the VEGFR‐3/VEGF‐C Complexes

**FIGURE 3:**
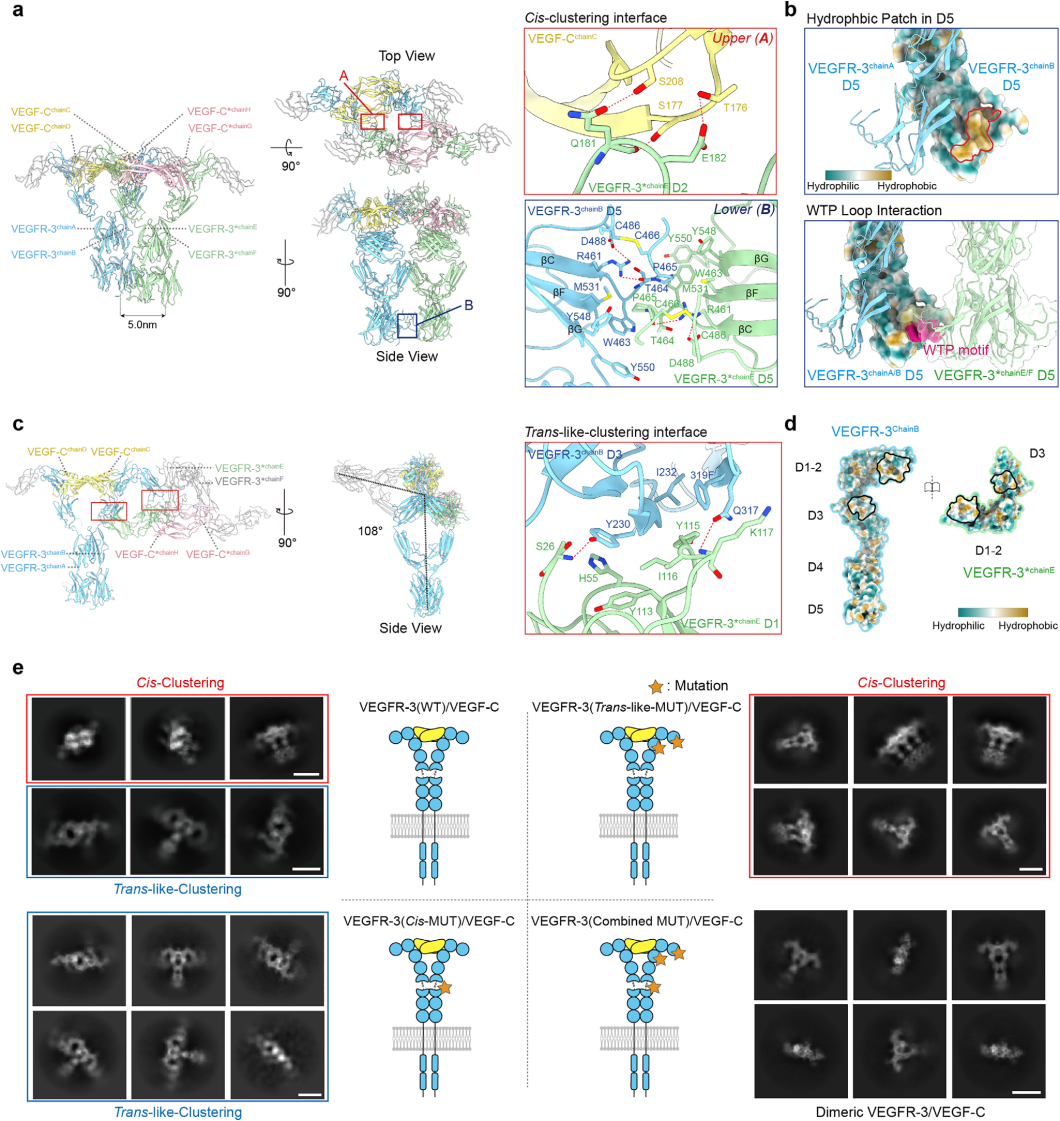
Structural analysis of the *cis*‐ and *trans*‐like‐clustered VEGFR‐3 ECD/VEGF‐C complexes. a Overall views (front, top, and side) of the *cis‐*clustered VEGFR‐3 ECD/VEGF‐C complex. The interfaces within Regions A and B involved in *cis‐*clustering are indicated (left), along with the close‐up views (right). The upper and lower interfaces correspond to the VEGFR‐3*^chainE^/VEGF‐C^chainC^ interactions (Region A, red box) and VEGFR‐3^chainB^ D5/VEGFR‐3*^chainE^ D5 interactions (Region B, blue box), respectively. b Hydrophobic surface potential of VEGFR‐3^chainB^ D5 domain within the *cis‐*clustered complex (top). The WTP loop mediating the D5–D5* hydrophobic interaction responsible for *cis‐*clustering is highlighted in magenta (bottom). c Overall views (front and side) of the *trans‐*like‐clustered VEGFR‐3 ECD/VEGF‐C complex. The symmetrical interfaces involved in *trans‐*like‐clustering are indicated by red boxes (left), with a close‐up view of the interaction interface between VEGFR‐3^chainB^ and VEGFR‐3*^chainE^ (right). a and c Unresolved domains are indicated with a gray ribbon, and the color scheme is identical to Figure 1d,e. In all close‐up views, the key interacting residues are represented by sticks and labeled. Hydrogen bonds and electrostatic interactions are indicated by red dashed lines. Secondary structure elements of VEGF‐C and VEGFR‐3 are labeled. d Hydrophobic surface potential of VEGFR‐3^chainB^ D1–D5 and VEGFR‐3*^chainE^ D1–D3 domains within the *trans*‐like‐clustered complex, shown in an open‐book view. Interaction interfaces in the D3 and D1* of VEGFR‐3 are outlined in black. e Representative 2D class averages of the VEGFR‐3 ECD/VEGF‐C complexes formed with wild‐type (WT) or clustering‐defective mutants (*cis‐*MUT, *trans*‐like‐MUT, and combined MUT). Schematic diagrams of WT and three mutant constructs are shown, with mutation sites marked by orange stars. Scale bar: 100 Å.

Structural analysis revealed that the dimeric unit, [VEGFR‐3/VEGF‐C]_2_, further assembles into higher‐order oligomers via two distinct clustering modes: *cis‐* and *trans*‐like‐clusters (Figure 3). In the *cis*‐clustering mode, [VEGFR‐3/VEGF‐C]_2_ are aligned laterally side‐by‐side with a spacing of ∼5.0 nm between the D5 membrane‐proximal regions of the two complexes. This assembly is stabilized by two primary interfaces (Figure 3a). The first is a symmetric “upper” interface (Region A), where the loop region of VEGFR‐3* D2 from the neighboring complex engages the β‐sheet of the VEGF‐C, burying ∼180 Å^2^ for each VEGFR‐3*^chainE^ D2/VEGF‐C^chainC^ and VEGFR‐3^chainB^ D2/VEGF‐C*^chainH^ complex; total ∼370 Å^2^ for the two Region A. These interactions are mediated by hydrogen bonds between the residues T176, S177, and S208 of VEGF‐C^chainC^ and the Q181 and E182 of VEGFR‐3*^chainE^; additionally, between T176, S177, and S208 of VEGF‐C*^chainH^ and the Q181 and E182 of VEGFR‐3^chainB^. The second is a “lower” interface (Region B), formed by reciprocal loops protruding from the D5s of adjacent receptors—VEGFR‐3^chainB^ and VEGFR‐3*^chainE^, burying ∼390 Å^2^ (Figure 3a, Region B, blue box). Here, the residues W463 and P465, located within the long loop connecting the βC and βD strands of D5, form a hydrophobic network with the nearby residues: M531, Y548, and Y550. This network interlocks with the corresponding loop of the neighboring receptor, shielding the hydrophobic patch and stabilizing the *cis*‐cluster conformation. We designated this critical W463–P465 structural element as the WTP motif, whose conformation is constrained by a C466–C486 disulfide bridge and electrostatic interactions involving R461 with D488 and the T464 main chain.

Sequence alignment revealed that the WTP motif is unique to VEGFR‐3 and is not observed with VEGFR‐1 or ‐2, although all VEGFR family members possess long and flexible loops within D5 spanning residues 464–495 (Figure S6). Notably, the corresponding region of the VEGFR‐2 contains three consecutive glutamate residues (EEE), which would likely induce electrostatic repulsion and prevent *cis*‐clustering. Furthermore, VEGFR‐3 harbors a unique proteolytic cleavage site, immediately downstream of the WTP motif. Critically, a truncation of this loop, or replacement with the corresponding sequence from VEGFR‐2, attenuates VEGFR‐3 signaling [23], underscoring the critical role of the WTP‐containing loop of D5 in receptor function (Figure 3b).

In contrast, the *trans*‐like‐clustering mode features an inverted, “head‐to‐tail” arrangement with an inter‐complex angle of ∼108° (Figure 3c). Such an assembly is mediated by reciprocal interactions between the D3 of one receptor and D1* of an adjacent receptor, burying ∼480 Å^2^ for each without the direct involvement of VEGF‐C. This interface is stabilized by aromatic ring packing and hydrophobic networks—Y230, I232, and F319 in VEGFR‐3^chainB^ D3 packing against H55, Y113, Y115, and I116 in VEGFR‐3*^chainE^ D1, and is reinforced by the surrounding electrostatic contacts: Y230 and Q317 in VEGFR‐3^chainB^ D3 with the main chains of S26 and K117 in VEGFR‐3*^chainE^ D1, respectively (Figure 3d; Figures S6 and Table S2). These residues are highly conserved across VEGFR‐3 orthologs but not among other VEGFR family members, suggesting the uniqueness of this *trans*‐like‐clustering arrangement to VEGFR‐3 (Figure S6).

To validate the significance of these interfaces for *cis‐* and *trans*‐like‐clustering, we designed structure‐guided mutants to selectively disrupt each clustering mode. To inhibit *cis*‐clustering, we replaced the hydrophobic WTP motif with three glutamate residues—*cis*‐MUT: W463E/T464E/P465E —mimicking the repulsion‐related sequence found in VEGFR‐2. To disrupt *trans*‐like‐clustering, we mutated key hydrophobic residues at the D3–D1* interface to alanine—*trans*‐like‐MUT: H55A/Y115A/Y230A. The resulting *cis‐*, *trans‐*like‐, and combined mutant VEGFR‐3/VEGF‐C complexes were purified and visualized via cryo‐EM 2D class averaging. Unlike the wild‐type (WT) complexes, which exhibited a mixture of *cis‐* and *trans‐*like‐clusters, the *cis*‐MUT complex failed to form the *cis*‐clusters and predominantly assembled into *trans*‐like‐clustered arrays; conversely, the *trans*‐like‐MUT complex mainly formed *cis*‐clusters (Figure 3e). Most importantly, the combined mutant (harboring the two sets of mutations) completely failed to oligomerize, existing solely as a discrete [VEGFR‐3/VEGF‐C]_2_ complex. These results demonstrate that the two clustering modes are structurally distinct and mutually exclusive, and that our structure‐guided mutations effectively and selectively ablated each interface without compromising the core dimeric structure.

### 2.5 Critical role of *cis*‐Clustering in VEGF‐C–Mediated VEGFR‐3 Signaling

**FIGURE 4:**
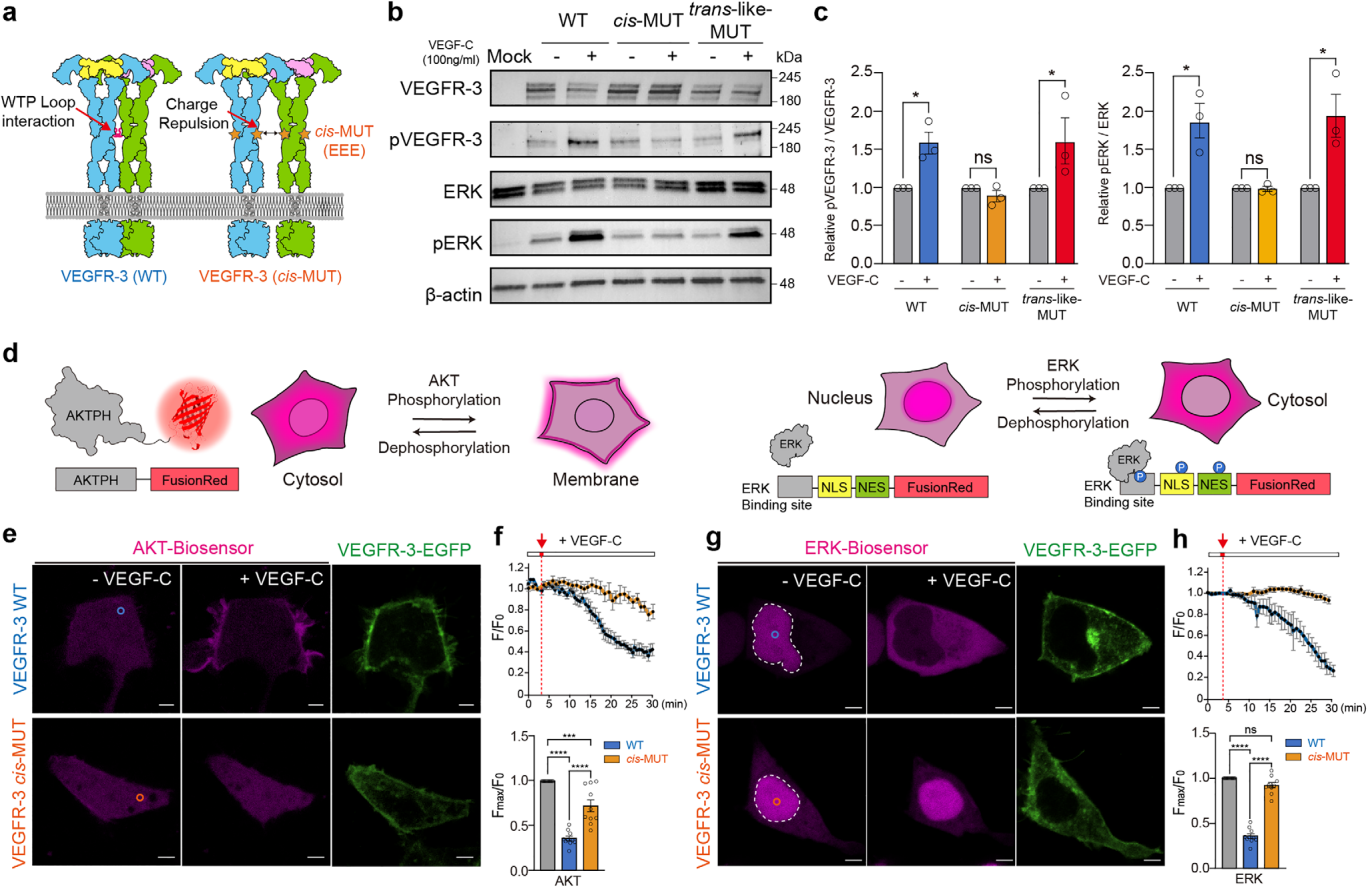
The *cis*‐clustering motif of VEGFR‐3 is critical for VEGF‐C‐mediated signaling. a Schematic diagram of the VEGFR‐3 wild‐type (WT) and *cis*‐clustering mutant (*cis*‐MUT) constructs. The *cis*‐clustering WTP motif (W463, T464, and P465) within the D5 domain is highlighted in magenta. For the *cis*‐MUT, these residues were substituted with negatively charged glutamate residues (W463E, T464E, and P465E) to induce charge repulsion (orange stars). b Immunoblot analysis of VEGFR‐3 and ERK phosphorylation in HEK293T cells transiently expressing VEGFR‐3 WT, *cis*‐MUT or *trans*‐like‐MUT following stimulation with 100 ng/ml VEGF‐C. c Quantification of immunoblot results presented in (b). Each data point represents an individual experiment (n = 3). Data were normalized to the levels of β‐actin (pVEGFR‐3/VEGFR‐3 or pERK/ERK) and are presented as mean ± SEM. Statistical significance was determined by a two‐tailed unpaired t‐test (ns, not significant; *p < 0.05 vs. without VEGF‐C stimulation). d Schematic diagrams illustrating the mode of action of the AKT‐ (AKTPH‐RFP, left) and the ERK biosensors (ERK‐KTR‐RFP, right). e and g Representative live‐cell images showing AKT (e) and ERK (g) activation in VEGFR‐3 WT or *cis*‐MUT‐expressing cells 25 min after treatment with 100 ng/ml VEGF‐C. VEGFR‐3‐EGFP images were acquired prior to VEGF‐C stimulation. Dashed lines indicate the nucleus, and colored circles mark regions of interest (ROI) for the quantification of the fluorescence intensities (F) of the AKT and ERK biosensors. Scale bars: 5 µm. f and h Time‐lapse quantification of AKT (f) and ERK (h) biosensors at ROI after VEGF‐C treatment (top). F was normalized to the baseline F_0_ at t = 0. VEGF‐C (100 ng/ml) was added at 2–3 min (red dashed line and arrow). Blue and orange lines represent fluorescence changes in the VEGFR‐3 WT‐ and *cis*‐MUT‐expressing cells, respectively. Data are the mean ± SEM (WT, n = 10; *cis*‐MUT, n = 10). Bar graphs present the F_max_/F_0_ ratio (bottom), where t = max is the time point with maximal intensity change. The gray bar indicates F_0_/F_0_. Each dot represents the data from an individual cell, and data are represented as the mean ± SEM (WT, n = 10; *cis*‐MUT, n = 10). Statistical significance was calculated using one‐way ANOVA with Tukey's multiple comparisons (ns, not significant; ****p < 0.0001, ***p < 0.001).

To assess the relevance of the clustering interfaces identified regarding cell function, we introduced the structure‐guided, clustering‐disruptive mutations (*cis*‐MUT: W463E/T464E/P465E; *trans*‐like‐MUT: H55A/Y115A/Y230A) into full‐length VEGFR‐3 (Figure 4a and Figure S7a). These constructs were transiently expressed in HEK293T cells, which lack an endogenous VEGFR‐3, enabling a direct comparison of the signal transduction between the WT VEGFR‐3 and clustering‐deficient receptors.

We examined the impact of VEGFR‐3 clustering on downstream signaling by employing two independent experimental strategies: immunoblotting for bulk population analysis and fluorescence‐based biosensors for real‐time, single‐cell monitoring. Serum‐starved cells expressing VEGFR‐3 WT, *cis*‐MUT, or *trans*‐like‐MUT were stimulated with 100 ng/ml VEGF‐C, and the phosphorylation levels of VEGFR‐3 and ERK were measured at 20 min via immunoblotting. Notably, WT and mutant VEGFR‐3 exhibited minimal basal VEGFR‐3 and ERK phosphorylation in the absence of VEGF‐C, indicating little ligand‐independent signaling under our experimental conditions. Surprisingly, despite VEGF‐C stimulation, VEGFR‐3 and ERK phosphorylation were markedly impaired in cells expressing the *cis*‐MUT receptor, whereas the *trans*‐like‐MUT receptor exhibited phosphorylation levels comparable to those of the WT (Figure 4b,c), indicating that the *cis*‐clustering of VEGFR‐3 is critical for VEGF‐C–dependent signal propagation.

As a complementary approach, we monitored the real‐time signaling dynamics at the single‐cell level in cells expressing VEGFR‐3 WT, *cis*‐MUT, or *trans*‐like‐MUT utilizing fluorescent biosensors. To analyze the intensity of AKT‐based signaling, we used a red fluorescent protein (RFP)‐fused AKT pleckstrin homology (PH) domain, which translocates to the plasma membrane (PM) upon VEGFR‐3‐mediated phosphoinositide 3‐kinase (PI3K) activation [31, 32]. For ERK signaling, we utilized an RFP‐conjugated Kinase Translocation Reporter (KTR) [33], composed of an ERK substrate sequence fused to nuclear localization and export signals (NLS and NES), which relocates from the nucleus to the cytoplasm upon its phosphorylation via ERK activation (Figure 4d). These biosensors were coexpressed with the EGFP‐tagged VEGFR‐3 constructs (WT or mutants) in HEK293T cells. Live‐cell imaging with the EGFP signal confirmed proper plasma membrane localization for all VEGFR‐3 constructs before stimulation (Figure 4e). Time‐lapse imaging was then performed before and after treatment with 100 ng/ml VEGF‐C.

In cells expressing VEGFR‐3 WT, VEGF‐C stimulation robustly triggered the translocation of the AKTPH sensor to the PM and the relocation of the ERK‐KTR sensor from the nucleus to the cytoplasm, indicating strong downstream signaling activation (Figure 4e‐h, top). In contrast, cells expressing the *cis*‐MUT showed no such discernible biosensor translocation (Figure 4e–h, bottom), consistent with the immunoblotting results above. Quantitative analysis confirmed that VEGFR‐3 WT induced markedly higher and more sustained activation of AKT and ERK (lasting for >20 min) compared to the VEGFR‐3 *cis*‐MUT (Figure 4f,h). Notably, cells expressing the *trans*‐like‐MUT did not show any signaling impairment, responding identically to the WT (Figure S7b, c). Taken together, these results establish that VEGF‐C‐induced *cis*‐clustering of VEGFR‐3 is critical for effective downstream signaling, whereas *trans*‐like‐clustering appears dispensable under our assay conditions.

### 2.6 Receptor Clustering as a Sufficient Driver of VEGFR‐3 Downstream Signaling

To test whether enforced receptor clustering can promote VEGFR‐3 signaling, we employed an optogenetic module comprising the light‐sensitive Cryptochrome 2 Photolyase Homology Region (CRY2PHR) domain derived from *Arabidopsis thaliana* [34]. CRY2PHR undergoes rapid and reversible oligomerization upon blue‐light exposure (450–500 nm), enabling the precise spatiotemporal control of protein clustering in mammalian cells [35, 36, 37]. This module is widely used to control clustering‐dependent pathways (e.g., RTKs and Wnt/β‐catenin), as induced proximity alone is often sufficient to trigger signal activation [35, 38]. Thus, CRY2PHR‐mediated optogenetic protein assembly would be an ideal approach for a systematic investigation of the impact of VEGFR‐3 clustering on downstream signaling.

**FIGURE 5:**
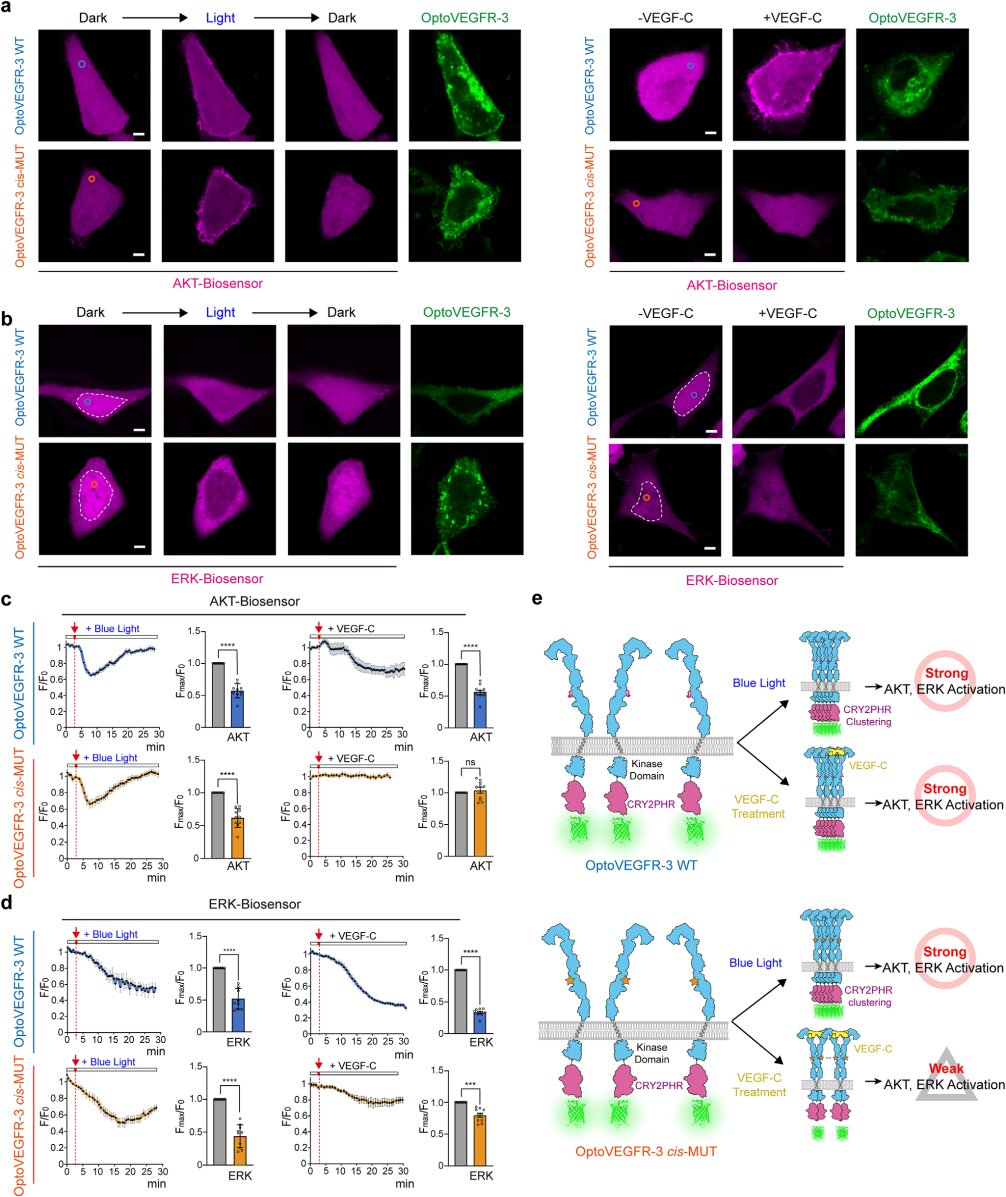
Optogenetically induced *cis*‐clustering of VEGFR‐3 activates downstream signaling. a and b Representative live‐cell images showing AKT (a) and ERK (b) activation in optoVEGFR‐3 WT or *cis*‐MUT‐expressing cells after exposure to blue light at 488 nm for 3 min (left) or stimulation with VEGF‐C (right). Live‐cell imaging was performed for 2–3 min before stimulation (Dark), followed by 3 min of blue‐light exposure (Light) and an additional 25 min of imaging after light withdrawal (Dark). optoVEGFR‐3 (EGFP) images were acquired prior to blue light or VEGF‐C stimulation. Dashed lines mark the nucleus, and colored circles indicate the regions of interest (ROIs) for fluorescence quantification. Scale bars: 5 µm. c and d Time‐lapse quantification of AKT (c) and ERK (d) biosensor fluorescence at ROIs after exposure to blue light at 488 nm for 3 min (left) or treatment with VEGF‐C (right). The stimulation point (blue‐light exposure or VEGF‐C treatment) is indicated by a red dotted line and arrow. Fluorescence intensity (F) was normalized to the baseline F_0_ at t = 0 min. Blue and orange lines represent fluorescence changes in optoVEGFR‐3 WT and *cis*‐MUT‐expressing cells, respectively. Data are presented as the mean ± SEM (WT, n = 10; *cis*‐MUT, n = 10). Bar graphs present the F_max_/F_0_ ratio (bottom), where t = max is the time point with maximal intensity change. The gray bar indicates F_0_/F_0_. Each dot represents the data from an individual cell, and data are represented as the mean ± SEM (WT, n = 10; *cis*‐MUT, n = 10). Data (F_max_/F_0_) are presented as mean ± SEM and statistical significance was determined by a two‐tailed unpaired t‐test (ns, not significant; ****P < 0.0001, ***P < 0.001). e Schematic illustration of the experimental design and results, comparing optoVEGFR‐3 activation and clustering by VEGF‐C treatment vs. optogenetic clustering in HEK293T cells transiently expressing optoVEGFR‐3 WT or *cis*‐MUT constructs.

To implement such a system, we generated an OptoVEGFR‐3 construct by fusing CRY2PHR and EGFP with the C‐terminus of full‐length VEGFR‐3 (WT or *cis‐*MUT) (Figure 5). This construct allows the real‐time visualization and light‐inducible oligomerization of the receptor, while retaining responsiveness to its natural ligand, VEGF‐C. HEK293T cells co‐expressing OptoVEGFR‐3 (WT or *cis‐*MUT) and the AKT/ERK biosensors were stimulated with either VEGF‐C or blue light (488 nm and 24 mW/cm^2^, every 30 s for a total duration of 2–3 min). As expected, the cells expressing OptoVEGFR‐3 WT robustly activated AKT and ERK signaling in response to either VEGF‐C or blue‐light stimulation, as evidenced by biosensor dynamics (Figure 5a–d). Consistent with the results in Figure 4, VEGF‐C induced sustained activation, while the light‐induced signal was transient and reversible, reflecting the known dynamics of CRY2PHR clustering [39].

Crucially, the OptoVEGFR‐3 *cis*‐MUT showed markedly impaired responses to VEGF‐C stimulation, confirming that VEGF‐C–dependent signaling requires the *cis*‐clustering interface. However, this defect was fully rescued by optogenetic clustering. Light‐induced oligomerization of the *cis‐*MUT receptor triggered robust AKT and ERK activation, with dynamics comparable to those of the light‐activated WT receptor.

These findings collectively demonstrate that receptor clustering is sufficient to drive robust VEGFR‐3 signal transduction. The ability of optogenetically enforced clustering to rescue the VEGFR‐3 *cis‐*MUT defect supports a model in which VEGF‐C‐induced receptor dimerization is followed by higher‐order *cis*‐assembly via the WTP loop, thereby promoting efficient signal amplification.

## 3 Discussion

In this study, we determined the cryo‐EM structure of the human VEGFR‐3 ECD in complex with VEGF‐C, revealing the mechanisms by which VEGF‐C binding drives canonical VEGFR‐3 dimerization and promotes further assembly into higher‐order oligomers—specifically, a lateral *cis‐*cluster and an inverted *trans‐*like‐cluster. Through this structural analysis combined with structure‐guided mutagenesis and optogenetic assays, we demonstrated that the *cis‐*clustering mediated by the unique WTP motif of D5 is essential for amplifying downstream signaling, whereas the *trans*‐like‐clustering interface appears dispensable for activation. These findings provide the first structural evidence for ligand‐induced receptor clustering within the VEGFR family member and establish a new paradigm for VEGFR‐3 activation beyond dimerization.

Our structural analysis of the canonical 2:2 hetero‐tetrameric unit in higher‐order assembly, resembling the conserved topology of other VEGFR/VEGF family members, provides crucial insights into the mechanisms of ligand recognition and receptor homotypic interaction (Figure 2a–c). Within this canonical 2:2 unit, the VEGF‐C dimer engages the VEGFR‐3 D2 domain through extensive hydrophobic contacts between one face of the β‐sandwich of VEGFR‐3 D2 and the β‐sheet located at the wing tip of the butterfly‐shaped VEGF‐C, involving the α‐βF loop, βA’, and βG of VEGFR‐3; and the β2, β4, and L3 of VEGF‐C. Within the resulting complementary hydrophobic cleft occurring between VEGFR‐3 D2 and VEGF‐C, the N‐terminal α1 helix of another VEGF‐C protomer is tightly intercalated, anchoring the ligand–receptor assembly. Moreover, L1 and L2 loops of each VEGF‐C protomer bind to the cleft formed by two β‐sheets of the VEGFR‐3 D3 β‐sandwich and the connecting loops via electrostatic interactions, while homotypic interactions in D5 stabilize the symmetric membrane‐proximal region through ionic and polar contacts.

Comparative structural analysis of our canonical [VEGFR‐3/VEGF‐C]_2_ complex with closely related family members—e.g., [VEGFR‐2 D2‐D3/VEGF‐C]_2_ (PDB ID 2X1W) [24] and [VEGFR‐1 D1‐D6/VEGF‐A]_2_ (PDB ID 5T89) [13]—provides profound insights into the structural determinants of ligand specificity and promiscuity. When VEGF ligands engage their receptors, specificity is governed primarily by the N‐terminal α1 helix, the hydrophobic β‐sheet surface at the “wing tip” of the butterfly‐shaped VEGF, and L1–L3 loops of VEGF, in coordination with the complementary architecture of the VEGFR D2–D3 domains. Although VEGF‐C binds with both VEGFR‐3 and VEGFR‐2, its affinity for VEGFR‐2 is modestly reduced. As detailed in our results (Figure 2g–h), this difference arises because the VEGFR‐2 D2 domain approaches the ligand at a steeper angle (∼15° more upright) and presents protruding hydrophobic residues (M197 and M213) that force a steric rotation of the key VEGF‐C α1 helix. This conformation results in less compact interfacial packing compared to the highly optimized and rigid interface of VEGFR‐3. In contrast, structural incompatibility precludes VEGF‐C from binding to VEGFR‐1. The D2 of VEGFR‐1 adopts an even steeper engagement angle than that of VEGFR‐2 (Figure S8a). Crucially, the long N‐terminal α1 helix of VEGF‐C—essential for high‐affinity anchoring—would severely clash with the protruding D1–D2 connecting loop of VEGFR‐1 (the “YSEIPE” sequence). Conversely, the corresponding region of VEGFR‐2 possesses a flexible linker (the “VSDGHG” sequence), and VEGFR‐3 features a shortened linker with a 6‐amino acid deletion and an outwardly displaced D2 βA’ strand. These structural adaptations specifically accommodate the long N‐terminal α1 helix of VEGF‐C (Figure S8b). Furthermore, VEGFR‐1 exhibits a unique D2–D3 juxtaposition where D3 is rotated relative to D2, creating additional steric hindrance for VEGF‐C L2 and L3 binding. These structural observations provide high‐resolution confirmation of earlier mosaic‐VEGF, VEGFR domain‐deletion/swap studies, which identified the extended N‐terminal helix of VEGF‐C/VEGF‐D as an important determinant of VEGFR‐3 recognition and VEGFR‐1 incompatibility [14, 40, 41], as well as VEGFR D2 as the principal determinant of ligand specificity [11]. Collectively, our structural insights elucidate the molecular basis by which VEGF‐C binds VEGFR‐3 with high affinity and VEGFR‐2 with modest affinity, while failing to bind and activate VEGFR‐1.

Consistent with previous structural studies of VEGF‐C/VEGFR complexes [14, 24], we used a C137A‐stabilized mature‐core VEGF‐C construct to ensure a stable and homogeneous receptor–ligand complex. Although our cryo‐EM structure clearly resolves the interchain C156–C165* disulfide bond (Figure S4c), we acknowledge that this covalently linked state may not fully represent the structural plasticity of native VEGF‐C, which can exist in non‐covalent or alternative redox‐dependent dimeric forms [26, 30, 42]. This distinction is critical for understanding receptor selectivity. For instance, the VEGF‐C C156S mutant selectively loses VEGFR‐2 activation while retaining VEGFR‐3 signaling [42, 43], implying that simple homodimerization is necessary but not sufficient for VEGFR‐2 engagement. These findings are consistent with the possibility that the C156–C165* disulfide linkage contributes to maintaining the precise dimer geometry or local conformational stability required for VEGFR‐2 engagement and activation, whereas VEGFR‐3 may exhibit greater tolerance for VEGF‐C dimerization plasticity. Future structural and biophysical studies incorporating native‐like non‐covalent assemblies and defined redox states will be important for resolving how these VEGF‐C dimerization dynamics govern VEGFR selectivity.

Notably, although previous biochemical studies suggested that VEGFR‐3 D1 may contribute to high‐affinity ligand binding together with D2 [41], the ligand‐contacting interface resolved in our cryo‐EM maps is primarily mediated by the D2–D3 domains, suggesting that these domains constitute the major structural anchors for VEGF‐C recognition. However, because VEGFR‐3 D1 is flexible and only partially resolved in our cryo‐EM map of the *trans*‐like‐clustered complex, our data do not exclude an auxiliary contribution of D1 or the D1–D2 junction to ligand engagement, potentially through stabilization of the D2–D3 architecture.

The discovery of *cis‐*clustering in VEGFR‐3 represents a significant advancement in our understanding of RTK activation (Figures 3, 4, 5). While VEGF‐induced dimerization has long been considered the “switch” for VEGFR activation, our data suggest that for VEGFR‐3, dimerization is necessary but insufficient for robust signaling; rather, higher‐order clustering acts as a critical amplifier. This finding aligns with emerging models for other RTKs, such as EGFR, Eph receptors, and Tie2, where oligomerization organizes kinases into a competent signaling geometry to enhance response fidelity and duration [16, 17, 44]. Indeed, the concept of higher‐order organization within the VEGF receptor family is not entirely new. Previous studies utilizing single‐molecule imaging and mathematical modeling have reported that VEGFR‐2 can display heterogeneous mobility and organize into high‐density nanodomains or clusters on the endothelial cell surface [45, 46, 47, 48]. However, unlike the pre‐formed dimers or direct transmembrane interactions observed in other RTKs [49, 50, 51], or the undefined clustering mechanisms inferred in prior studies on VEGFR, our work clearly demonstrated that VEGFR‐3 *cis‐*clustering is uniquely driven by a distinct extracellular loop in D5, the WTP motif. This observation highlights a novel assembly mechanism specific to the VEGFR‐3 that may facilitate the integration of weak ligand signals into a robust cellular response. The CRY2PHR‐based optogenetic system was used as a mechanistic tool to test signaling driven by enforced receptor clustering, while the stoichiometry and spatial organization of native VEGFR‐3 assemblies remain to be established.

In addition to *cis*‐clustering, our cryo‐EM analysis revealed an inverted *trans*‐like assembly of the VEGFR‐3/VEGF‐C ectodomain complex. However, its compatibility with a rigid cell–cell contact model and its physiological relevance remain to be fully validated. Although the substantial flexibility of the D3–D4 linker (Figure 2f) and the membrane‐proximal segments may allow the VEGFR‐3 ectodomain to accommodate multiple orientations, our current study does not provide direct evidence of *trans*‐like‐clustering between opposing cell membranes in vivo. Functionally, the *trans*‐like‐MUT receptor retained VEGF‐C‐induced VEGFR‐3, AKT, and ERK activation, indicating that this interface is dispensable for canonical VEGF‐C‐induced signaling under our assay conditions. Nevertheless, these results do not exclude the possibility that *trans*‐like interactions may have context‐dependent or noncanonical functions, such as contributing to junctional architecture and the maintenance of lymphatic endothelial integrity. This possibility is conceptually reminiscent of the endothelial receptor Tie2, which forms distinct Ang1‐bridged trans‐associated complexes at cell–cell contacts to promote vascular quiescence and stability [52, 53]. Similarly, EphA2 receptors have been shown to assemble into higher‐order clusters at cell–cell interfaces, where the degree of clustering dictates the balance between cell adhesion and repulsion [20, 54]. Moreover, since our current study did not include the co‐receptor Neuropilin‐2 (NRP2) and used a mature‐core VEGF‐C construct comprising residues 103–215, which lacks the C‐terminal NRP2‐interacting region containing Arg223 [55], we cannot determine whether co‐receptors further stabilize or modulate *cis‐* or inverted *trans*‐like VEGFR‐3 assemblies. Given that the higher‐order assemblies were characterized using purified VEGFR‐3 ectodomain/VEGF‐C complexes at concentrations required for structural analysis, while the functional assays were performed in transiently transfected HEK293T cells, these findings support a functional role for the *cis*‐clustering interface in VEGFR‐3 signaling under the conditions tested. Future investigations employing physiologically processed VEGF‐C, co‐receptors, and more complex cellular systems or in vivo mouse models will be required to resolve the physiological relevance and diverse functional outcomes of these distinct clustering modes.

While anti‐angiogenic therapies targeting VEGF signaling, such as bevacizumab (Avastin) [56] and ranibizumab (Lucentis) [57], have been successfully implemented for treating various cancers and retinal diseases, no FDA‐approved drugs currently target lymphangiogenesis. Despite growing evidence of its role in cancer progression, candidates targeting the VEGF‐C/VEGFR‐3 pathway—such as anti‐VEGF‐C antibodies (VGX‐100 [58] for glioblastoma), VEGF‐C/D traps (OPT‐302 [59] for neovascular eye disease), and selective VEGFR‐3 inhibitors (EVT801 [60] for solid tumors)— have been evaluated in clinical trials, underscoring the therapeutic interest in this pathway. In this context, our findings offer a structural reinterpretation of the mechanism underlying the inhibitory function of the anti‐VEGFR‐3 antibody. Previously, 2E11 was shown to bind a conformational epitope of D5 and inhibit ligand‐induced activation, notably synergizing with ligand‐blocking antibodies (e.g., 3C5) to suppress downstream signaling and angiogenic sprouting [23]. However, our structural analysis suggests that this epitope is located close to the *cis‐*clustering interface (the WTP loop) with 2E11, most likely acting as a “cluster‐breaker” and sterically hindering the formation of higher‐order *cis‐*clusters. This finding defines a novel class of allosteric inhibitors that may offer superior efficacy in tumors with high local concentrations of VEGF‐C, where competitive inhibitors targeting ligand‐binding sites often fail. Conversely, our optogenetic rescue data suggest that synthetic “molecular staples” mimicking the *cis‐*cluster could serve as potent agonists for treating hereditary lymphedema. Future studies leveraging our high‐resolution structure and applying AI‐driven methods to design proteins, such as specific‐epitope targeting antibody or de novo mini‐binders, to generate candidates that precisely target these clustering interfaces will be essential. These candidates will not only serve to further validate the physiological significance of clustering in in vivo models but also to evaluate the therapeutic potential of clustering blockade as a novel modality for suppressing pathological lymphangiogenesis in cancer metastasis and other vascular diseases.

In summary, our work unveils the structural plasticity of the VEGFR‐3 ECD and establishes *cis‐*clustering as a key mechanism for signal amplification. This paradigm shift not only deepens our understanding of the VEGFR/VEGF complex but also provides a precise structural blueprint for developing next‐generation therapeutics targeting lymphatic vascular diseases.

## 4 Experimental Section

### 4.1 Cell Lines and Cell Culture

Expi293F cells (#A14527; Thermo Fisher Scientific) were cultured in Expi293 expression medium (#A1435102, Thermo Fisher) using a humidified shaking incubator at 37°C and under 8% CO_2_. HEK293T cells (#CRL‐3216, ATCC) were cultured in Dulbecco's Modified Eagle Medium (DMEM; #LM 001–05, Welgene Inc.) supplemented with 10% fetal bovine serum (FBS; #12484020, Thermo Fisher Scientific) and 1% antibiotic–antimycotic (#15240062, Thermo Fisher Scientific) at 37°C in a humidified incubator under 5% CO_2_.

### 4.2 Constructs for Recombinant Protein Expression and Mutagenesis

The gene encoding the human VEGFR‐3 ECD (residues 1–798; #HG29697‐CF, Sino Biological Inc.) was subcloned into the modified pcDNA3.1 vector (#V86020, Addgene) containing a C‐terminal 3C protease cleavage site followed by an Fc tag. The DNA encoding an N‐terminal signal peptide followed by human VEGF‐C residues (residues 103–215; #KU036380, Korea Human Gene Bank) [14, 30] was subcloned into the modified pcDNA3.1 vector (#V86020, Addgene) with a C‐terminal 6x His tag. A C137A mutation was introduced into this VEGF‐C construct by site‐directed mutagenesis to enhance expression efficiency, as previously described [25]. The N‐terminal signal peptide enables secretion of recombinant VEGF‐C into the culture medium and is expected to be cleaved during the secretory pathway, leaving only minimal vector‐derived residues at the cloning junctions. The recombinant VEGF‐C protein derived from this construct (residues 103–215 with a C‐terminal 6x His tag) was used for cryo‐EM sample preparation and all cell‐based functional assays, including Western blotting and live‐cell imaging. For live‐cell imaging, the full‐length human VEGFR‐3 (residues 1–1363) was subcloned into the NheI and BamHI sites of the modified pEGFP‐N1 vector, fusing it to a C‐terminal flexible (Gly_4_Ser)_2_ linker followed by EGFP. C‐terminally fluorescent‐tagged VEGFR constructs have previously been used for live‐cell analysis of receptor localization and trafficking, including VEGFR‐2‐EGFP in HEK293T cells and VEGFR‐3‐GFP in HUVECs [61, 62]. For optogenetic clustering of the full‐length VEGFR‐3, the CRY2PHR (Cryptochrome 2 Photolyase Homology Region) domain (residues 1–498) was inserted between VEGFR‐3 and the EGFP‐encoding sequence within the full‐length VEGFR‐3 construct. Clustering‐deficient mutants were generated from the full‐length VEGFR‐3 construct using site‐directed mutagenesis and overlap PCR. These included the *cis*‐ (W463E/T464E/P465E), *trans*‐like‐ (H55A/Y115A/Y230A), and combined (W463E/T464E/P465E/H55A/Y115A/Y230A) mutants. All subcloning was performed using the EZ‐Fusion HT cloning kit (#EZ015TM, Enzynomics), and the primer sequences employed are listed in Table S4.

### 4.3 Expression and Purification of Recombinant Proteins

The human VEGFR‐3 ECD/VEGF‐C complex was produced by transient co‐transfection of Expi293F cells (2 × 10^6^ cells/ml) with plasmids encoding the VEGFR‐3 ECD‐Fc and VEGF‐C‐6x His using the ExpiFectamine 293 Transfection Kit (#A14524, Thermo Fisher Scientific). Cells were cultured at 37°C and under 8% CO_2_ with orbital shaking at 125 rpm for 3 days. Culture supernatants were harvested by centrifugation, and the secreted VEGFR‐3‐Fc/VEGF‐C complex was purified by affinity chromatography using Protein A resin (#1010100, Amicogen) equilibrated with PBS. After washing with 10 column volumes of PBS, the Fc tag was removed by on‐resin cleavage with 3C protease (#88346, Thermo Fisher Scientific) overnight at 4°C. The cleaved VEGFR‐3/VEGF‐C complex was eluted with five column volumes of PBS, concentrated using an Amicon Ultra centrifugal filter (#UFC9100, Millipore), and further purified by SEC on a Superdex 200 Increase 10/300 GL column (#GE28‐9909‐44, Cytiva) pre‐equilibrated with PBS. Peak fractions corresponding to the VEGFR‐3 ECD/VEGF‐C complex were pooled, concentrated, and used for negative‐stain and cryo‐EM analyses. Protein purity was assessed by SDS‐PAGE, and concentration was determined utilizing the Bradford assay kit (#23238, Thermo Fisher Scientific).

For cell‐based functional assays, recombinant VEGF‐C was purified separately. VEGF‐C‐6x His (residues 103–215, carrying the C137A substitution) was expressed in Expi293F cells under the same transfection and culture conditions. After 3 days, culture supernatants were harvested by centrifugation and applied to Ni‐NTA resin (#30230, Qiagen) equilibrated with PBS. The resin was washed with PBS containing 30 mM imidazole, and bound VEGF‐C was eluted with PBS containing 250 mM imidazole. Eluted fractions were concentrated, further purified by SEC on a Superdex 200 Increase 10/300 GL column pre‐equilibrated with PBS, aliquoted, snap‐frozen, and stored at −80°C until use.

### 4.4 Negative Staining and Image Processing for 2D Class Averages

The VEGFR‐3 ECD/VEGF‐C complex was diluted to 14.5 µg/ml in PBS, and 2.5 µL of the dilution was applied to a glow‐discharged Carbon Film 300 Mesh copper grid (#CF300‐Cu‐50, Electron Microscopy Sciences). After 1 min, the grid was blotted, washed twice with 20 µL of distilled water, and stained with 20 µL of 0.76% uranyl formate for 1 min. After blotting excess stain, the grid was air‐dried. Electron micrographs were acquired at ×52,000 magnification on a Tecnai G2 Spirit microscope (FEI, Thermo Fisher Scientific) operating at 120 kV, equipped with a 4K × 4K Eagle camera (FEI). Images were collected at a defocus of –1.8 µm and an electron dose of 30 e^−^/Å^2^, yielding a pixel size of 2.11 Å. Micrographs were imported into cryoSPARC [63], and contrast transfer function (CTF) parameters were estimated using patch CTF estimation. A total of 9,799 particles from 64 micrographs were manually picked, extracted (box size: 256×256 pixels), and subjected to 2D classification.

### 4.5 Cryo‐EM Sample Preparation and Data Collection

Quantifoil R 1.2/1.3 200 Mesh, holey carbon copper grid (#Q2100CR1.3, Electron Microscopy Sciences) was glow‐discharged for 90 s at 15 mA using a PELCO easiGlow system (Ted Pella). Then, 3 µl of the purified VEGFR‐3/VEGF‐C complex (∼1 mg/ml) was applied to the grid at 4°C and under 100% humidity. The grid was blotted for 3 s and plunge‐frozen in liquid ethane using a Vitrobot MkIV system (Thermo Fisher Scientific). Micrographs were acquired on a Titan Krios G4 TEM (Thermo Fisher Scientific) operating at 300 keV, equipped with a K3 direct electron detector (Gatan Inc.) and a GIF‐quantum energy filter (slit width: 20 eV) at the Institute for Basic Science (IBS). Automated data collection was performed using EPU software at a calibrated magnification of ×105,000 in single‐electron counting and correlated double sampling (CDS) mode, resulting in pixel sizes of 0.848, 0.849, and 0.828 Å/pixel for the *cis*‐clustering WT,  *trans*‐like‐clustering WT, and the *cis*/*trans‐like*/combined mutant datasets, respectively.

For the *cis*‐clustering WT dataset, a total of 8,732 movies were collected (defocus range: −0.7 to −1.9 µm). An initial set of 2,000 movies revealed a significant orientation bias, with top/bottom views dominating (>50% of 2D class averages). To obtain side‐view for improved angular distribution, subsequent data were collected at 15° (3,167 movies) and 25° (5,565 movies) tilt angles. Each micrograph was dose‐fractionated into 56 frames under a dose rate of 8.1 e^−^/pixel/s with a total exposure time of 5.98 s, resulting in a total dose of ∼67.3 e^−^/Å[2]. For the *trans*‐like‐clustering dataset, a total of 11,708 movies were collected (defocus range: −0.7 to −2.2 µm). Each micrograph was dose‐fractionated into 54 frames under a dose rate of 8.1 e^−^/pixel/s with a total exposure time of 5.77 s, resulting in a total dose of ∼64.8 e^−^/Å[2]. For the *cis‐*, *trans‐like*, or combined mutant datasets, a total of 1,829 movies (*cis*‐mutant), 2,496 movies (*trans*‐like‐mutant), and 2,496 movies (combined mutant) were collected (defocus range: −0.7 to −1.8 µm). Each micrograph was dose‐fractionated into 51 frames under a dose rate of 8.4 e^−^/pixel/s with a total exposure time of 5.45 s, resulting in a total dose of ∼66.8 e^−^/Å^2^. The mutant datasets were used exclusively for 2D class averaging. Detailed image acquisition parameters are summarized in Table S1.

### 4.6 Image Processing, Model Building, and Refinement

The detailed image processing workflow and statistics are summarized in Figures S2d, S3c, Table S1. All processing was performed using cryoSPARC v.3.3.1 [63]. Micrographs were subjected to patch motion correction [64] and patch CTF estimation [65].

For the *cis*‐clustering dataset (8,732 movies from 15° and 25° tilts), particles were picked applying a template‐based approach. A total of 4,041,738 initial extracted particles (2,919,230 and 1,122,508 particles from the 25° and 15° datasets, respectively) were processed through 2D classification, multi‐class ab initio reconstruction, and heterogeneous refinement in cryoSPARC for dataset clean‐up. The resulting 1,087,789 particles were re‐extracted (box size: 400×400 pixels) and subjected to further ab initio reconstruction and heterogeneous refinement.

For the *trans*‐like‐clustering dataset (11,708 movies), 1,495,805 particles were initially picked from the randomly chosen 9,267 micrographs using a blob‐based particle picker in cryoSPARC. Representative 2D classes were used as templates for subsequent template‐based particle picking from the full datasets. A total of 2,217,730 initial extracted particles (box size: 400×400 pixels) were binned (2x) and excluded bad particles through 2D classification, multi‐class ab initio reconstruction, and heterogeneous refinement in cryoSPARC. The resulting 965,510 particles were then unbinned (box size: 400 × 400 pixels) and subjected to further ab initio reconstruction and heterogeneous refinement.

The final particles for *cis*‐clustering (580,022 particles) and *trans*‐like‐clustering (486,815 particles), selected from 3D classes displaying well‐defined secondary structures, were subjected to non‐uniform refinement with per‐particle defocus and per‐group CTF optimization, obtaining a final map at overall resolutions of 3.3 and 3.2 Å, respectively (with a tight mask). Final map resolutions were based on the gold‐standard Fourier shell correlation (FSC) = 0.143 criterion [66, 67], and local resolutions were estimated by Blocres [68].

For the mutant datasets (*cis‐*, *trans‐like*, or combined mutant), particles were picked using both blob‐ and template‐based methods. The resulting particle sets (510,332 for *cis‐*mutant; 218,915 for *trans*‐like‐mutant; 610,088 for combined mutant) were subjected to 2D classification. These mutant datasets were utilized only for 2D class averaging to confirm their respective oligomeric states.

Model building was performed using maps post‐processed with DeepEMhancer [69], into which the reported crystal structures (VEGFR‐3 D1‐D2/VEGF‐C, PDB ID 4BSK; and VEGFR‐3 D4‐D5, PDB ID 4BSJ) along with an Alphafold3‐predicted structure of the VEGFR‐3 D3 domain [70], were docked. Then, the models were manually adjusted in Coot (v.0.8.9.2) [71] and subjected to real‐space refinement using the Phenix package [72]. Refinement statistics are summarized in Table S1. All figures for cryo‐EM maps and structural models were generated using ChimeraX (v.1.3) [73].

### 4.7 Immunoblotting for Analysis of VEGFR‐3 Signal Activation

HEK293T cells in 60‐mm dishes were transfected at 70%–80% confluency with the full‐length VEGFR‐3‐EGFP constructs (WT, *cis‐*mutant, or *trans*‐like‐mutant) using Lipofectamine 2000 (#11668027, Invitrogen) according to the manufacturer's protocol. Following 8 h of incubation in a serum‐free medium, the medium was replaced with a complete medium, and cells were cultured for an additional 3–4 days. Prior to stimulation, cells were serum‐starved for 1 h, then stimulated with 100 ng/ml human VEGF‐C in a serum‐free medium for 20 min. Cells were harvested and lysed in 200 µL of lysis buffer (10 mM Tris‐Cl pH 7.4, 150 mM NaCl, 5 mM EDTA, 10% glycerol, and 1% Triton X‐100) supplemented with protease (#4906845001, Roche) and phosphatase (#11836170001, Roche) inhibitors. Lysates were clarified by centrifugation at 15,000 g and 4°C for 15 min, and protein concentrations were determined using the Bradford assay. Equal amounts of cell lysates (20–30 µg of proteins) were separated on a 4–20% SDS‐PAGE gel (#4561094, Bio‐Rad Laboratories) under reducing conditions and transferred to a 0.45‐µm PVDF membrane. Membranes were blocked with 5% (w/v) skim milk in TBS‐T for 1 h at room temperature and incubated overnight at 4°C with primary antibodies (1:1000 dilution). The following primary antibodies were used: anti‐AKT (rabbit, #9272, Cell Signaling), anti‐p‐AKT (rabbit, #9271, Cell Signaling), anti‐p‐ERK (rabbit, #4370, Cell Signaling), anti‐ERK (rabbit, #9102, Cell Signaling), anti‐β‐actin (C4) (mouse, #sc‐47778, Santa Cruz Biotechnology, Inc.), anti‐p‐hVEGFR‐3 (rabbit, 120232, Covalab), and anti‐hVEGFR‐3 (goat, #AF349, R&D Systems). After washing, the membranes were incubated with horseradish peroxidase (HRP)‐conjugated secondary antibodies (1:5000 dilution) for 1 h at room temperature. The appropriate HRP‐conjugated secondary antibodies used were: anti‐rabbit IgG (#7074, Cell Signaling), anti‐goat IgG (#HAF109, R&D), and anti‐mouse IgG (#7076, Cell Signaling). Immunoreactive bands were visualized using the EzWestLumiOne chemiluminescence detection reagent (#2332632, ATTO).

### 4.8 Live‐Cell Imaging and Data Processing

HEK293T cells were seeded onto µ‐Plate 96 Well square plates (#89626, ibidi) pre‐coated with poly‐D‐lysine to enhance cell attachment. At 70%–80% confluency, cells were co‐transfected with the full‐length VEGFR‐3‐EGFP constructs (WT, *cis‐*mutant, or *trans*‐like‐mutant) and a signaling biosensor (AKT or ERK biosensor) using Lipofectamine 2000 (#11668027, Invitrogen). After 48 h post‐transfection, the cells were serum‐starved for at least 4 h before imaging. For live‐cell imaging, the cells were stimulated with 100 ng/ml of VEGF‐C and imaged every 25 s, starting 2–3 min before stimulation and continuing for 25 min post‐stimulation. Images were acquired on a Nikon Ti‐E Eclipse PFS confocal microscope (x60 objective) with 488 nm and 561 nm lasers to monitor the VEGFR‐3‐EGFP and RFP‐labeled biosensors, respectively. Environmental conditions were maintained at 37°C and 5% or 10% CO_2_ using a Chamlide TC system (Live Cell Instruments).

For optogenetic control, the cells were co‐transfected with VEGFR‐3‐CRY2PHR‐EGFP constructs (WT or *cis‐*mutant) and biosensors as described above. Instead of ligand stimulation, blue light activation (488 nm laser) was performed with pulses applied every 30 s for a total duration of 2–3 min.

For quantification of ERK and AKT activation, time zero (t = 0) was defined as the frame immediately preceding stimulation. All fluorescence intensity values were normalized to the intensity at t = 0 (F/F_0_). To compare activation levels, the maximal intensity change (F_max_/F_0_) was determined and plotted.

### 4.9 Quantification and Statistical Analysis

The data reported in figures and tables are the mean ± Standard Error of the Mean (SEM). Data quantification was performed using ImageJ, and statistical analyses were conducted using GraphPad Prism v10.3.1. The number of biological and technical replicates (n), statistical methods, and thresholds are specified in the figure legends.

## Author Contributions

R.C., J.A., S.L., and H.M.K. designed the experiments and analyzed the data; R.C. and J.A. determined the cryo‐EM structure; D.S.L. purified the proteins; J.Y., G.H., R.C., carried out the immunoblotting assay and live‐cell imaging; and R.C., J.A., S.L., and H.M.K. wrote the manuscript.

## Conflicts of Interest

The authors declare no conflicts of interest.

## Supporting information




**Supporting File 1**: advs77728‐sup‐0001‐SuppMat.pdf.

**Supporting File 2**: advs77728‐sup‐0002‐SuppMat.pdf.

## Data Availability

The atomic coordinates and cryo‐EM maps for the *cis‐* and *trans*‐like‐clustered VEGFR‐3 ECD/VEGF‐C complexes have been deposited to the Protein Data Bank and Microscopy Data Bank. The accession numbers for each complex are as follows: PDB 21ES and EMD‐67618 for the *cis‐*clustered VEGFR‐3 ECD/VEGF‐C complex, and PDB 21EI and EMD‐67611 for the *trans*‐like‐clustered VEGFR‐3 ECD/VEGF‐C complex. All other data are available in the manuscript or the Supplementary Information. Any other data supporting the findings of this study are available from the corresponding author (hm_kim@kaist.ac.kr), which can be provided upon reasonable request. Source data are provided with this paper.

## References

[1] M. Lohela , M. Bry , T. Tammela , and K. Alitalo , “VEGFs and receptors involved in angiogenesis versus lymphangiogenesis,” Current Opinion in Cell Biology 21, no. 2 (2009): 154–165, 10.1016/j.ceb.2008.12.012. 19230644

[2] M. Simons , E. Gordon , and L. Claesson‐Welsh , “Mechanisms and regulation of endothelial VEGF receptor signalling,” Nature Reviews Molecular Cell Biology 17, no. 10 (2016): 611–625, 10.1038/nrm.2016.87. 27461391

[3] K. Alitalo , “The lymphatic vasculature in disease,” Nature Medicine 17, no. 11 (2011): 1371–1380, 10.1038/nm.2545. 22064427

[4] P. Lorenc , A. Sikorska , S. Molenda , N. Guzniczak , H. Dams‐Kozlowska , and A. Florczak , “Physiological and tumor‐associated angiogenesis: Key factors and therapy targeting VEGF/VEGFR pathway,” Biomedicine & Pharmacotherapy 180 (2024): 117585, 10.1016/j.biopha.2024.117585. 39442237

[5] C. S. Lee , M. J. Kim , A. Kumar , H. W. Lee , Y. L. Yang , and Y. Kim , “Vascular endothelial growth factor signaling in health and disease: From molecular mechanisms to therapeutic perspectives,” Signal Transduction and Targeted Therapy 10, no. 1 (2025): 170, 10.1038/s41392-025-02249-0. 40383803 PMC12086256

[6] A. Szuba , M. Skobe , M. J. Karkkainen , et al., “Therapeutic lymphangiogenesis with human recombinant VEGF‐C,” The FASEB Journal 16, no. 14 (2002): 1985–1987, 10.1096/fj.02-0401fje. 12397087

[7] M. Lähteenvuo , K. Honkonen , T. Tervala , et al., “Growth factor therapy and autologous lymph node transfer in lymphedema,” Circulation 123, no. 6 (2011): 613–620, 10.1161/CIRCULATIONAHA.110.965384. 21282502

[8] D. Szoke , G. Kovács , É. Kemecsei , et al., “Nucleoside‐modified VEGFC mRNA induces organ‐specific lymphatic growth and reverses experimental lymphedema,” Nature Communications 12, no. 1 (2021): 3460, 10.1038/s41467-021-23546-6. PMC8187400 34103491

[9] M. Shibuya , “VEGFR and type‐V RTK activation and signaling,” Cold Spring Harbor Perspectives in Biology 5, no. 10 (2013): a009092–a009092, 10.1101/cshperspect.a009092. 24086040 PMC3783052

[10] J. Kunnapuu , H. Bokharaie , and M. Jeltsch , “Proteolytic Cleavages in the VEGF Family: Generating Diversity among Angiogenic VEGFs, Essential for the Activation of Lymphangiogenic VEGFs,” Biology (Basel) 10 (2021): 167, 10.3390/biology10020167. 33672235 PMC7926383

[11] T. Davis‐Smyth , H. Chen , J. Park , L. G. Presta , and N. Ferrara , “The second immunoglobulin‐like domain of the VEGF tyrosine kinase receptor Flt‐1 determines ligand binding and may initiate a signal transduction cascade,” The EMBO Journal 15, no. 18 (1996): 4919–4927. 8890165 PMC452229

[12] B. Barleon , F. Totzke , C. Herzog , et al., “Mapping of the sites for ligand binding and receptor dimerization at the extracellular domain of the vascular endothelial growth factor receptor FLT‐1,” Journal of Biological Chemistry 272, no. 16 (1997): 10382–10388, 10.1074/jbc.272.16.10382. 9099677

[13] S. Markovic‐Mueller , E. Stuttfeld , M. Asthana , et al., “Structure of the Full‐length VEGFR‐1 Extracellular Domain in Complex with VEGF‐A,” Structure 25, no. 2 (2017): 341–352, 10.1016/j.str.2016.12.012. 28111021

[14] V.‐M. Leppänen , D. Tvorogov , K. Kisko , et al., “Structural and mechanistic insights into VEGF receptor 3 ligand binding and activation,” Proceedings of the National Academy of Sciences 110, no. 32 (2013): 12960–12965, 10.1073/pnas.1301415110. PMC3740881 23878260

[15] A. K. Olsson , A. Dimberg , J. Kreuger , and L. Claesson‐Welsh , “VEGF receptor signalling ? in control of vascular function,” Nature Reviews Molecular Cell Biology 7, no. 5 (2006): 359–371, 10.1038/nrm1911. 16633338

[16] Y. Wang , J. Gao , X. Guo , et al., “Regulation of EGFR nanocluster formation by ionic protein‐lipid interaction,” Cell Research 24, no. 8 (2014): 959–976, 10.1038/cr.2014.89. 25001389 PMC4123299

[17] J. P. Himanen , L. Yermekbayeva , P. W. Janes , et al., “Architecture of Eph receptor clusters,” Proceedings of the National Academy of Sciences 107, no. 24 (2010): 10860–10865, 10.1073/pnas.1004148107. PMC2890748 20505120

[18] S. I. Liang , B. van Lengerich , K. Eichel , et al., “Phosphorylated EGFR Dimers Are Not Sufficient to Activate Ras,” Cell Reports 22, no. 10 (2018): 2593–2600, 10.1016/j.celrep.2018.02.031. 29514089 PMC5916813

[19] Y. Huang , S. Bharill , D. Karandur , et al., “Molecular basis for multimerization in the activation of the epidermal growth factor receptor,” Elife 5 (2016): e14107, 10.7554/eLife.14107. 27017828 PMC4902571

[20] E. Seiradake , K. Harlos , G. Sutton , A. R. Aricescu , and E. Y. Jones , “An extracellular steric seeding mechanism for Eph‐ephrin signaling platform assembly,” Nature Structural & Molecular Biology 17, no. 4 (2010): 398–402, 10.1038/nsmb.1782. PMC3672960 20228801

[21] J. Lee , A. Gray , J. Yuan , S. M. Luoh , H. Avraham , and W. I. Wood , “Vascular endothelial growth factor‐related protein: A ligand and specific activator of the tyrosine kinase receptor Flt4,” Proceedings of the National Academy of Sciences 93, no. 5 (1996): 1988–1992, 10.1073/pnas.93.5.1988. PMC39896 8700872

[22] K. Pajusola , O. Aprelikova , G. Pelicci , H. Weich , L. Claesson‐Welsh , and K. Alitalo , “Signalling properties of FLT4, a proteolytically processed receptor tyrosine kinase related to two VEGF receptors,” Oncogene 9 (1994): 3545–3555. 7970715

[23] D. Tvorogov , A. Anisimov , W. Zheng , et al., “Effective suppression of vascular network formation by combination of antibodies blocking VEGFR ligand binding and receptor dimerization,” Cancer Cell 18, no. 6 (2010): 630–640, 10.1016/j.ccr.2010.11.001. 21130043

[24] V.‐M. Leppänen , A. E. Prota , M. Jeltsch , et al., “Structural determinants of growth factor binding and specificity by VEGF receptor 2,” Proceedings of the National Academy of Sciences 107, no. 6 (2010): 2425–2430, 10.1073/pnas.0914318107. PMC2823880 20145116

[25] A. Anisimov , A. Alitalo , P. Korpisalo , et al., “Activated forms of VEGF‐C and VEGF‐D provide improved vascular function in skeletal muscle,” Circulation Research 104, no. 11 (2009): 1302–1312, 10.1161/CIRCRESAHA.109.197830. 19443835 PMC2776655

[26] J. Chiu , J. W. Wong , M. Gerometta , and P. J. Hogg , “Mechanism of dimerization of a recombinant mature vascular endothelial growth factor C,” Biochemistry 53, no. 1 (2014): 7–9, 10.1021/bi401518b. 24354278

[27] C. Ruch , G. Skiniotis , M. O. Steinmetz , T. Walz , and K. Ballmer‐Hofer , “Structure of a VEGF–VEGF receptor complex determined by electron microscopy,” Nature Structural & Molecular Biology 14, no. 3 (2007): 249–250, 10.1038/nsmb1202. 17293873

[28] S. Iyer , P. I. Darley , and K. R. Acharya , “Structural Insights into the Binding of Vascular Endothelial Growth Factor‐B by VEGFR‐1D2,” Journal of Biological Chemistry 285, no. 31 (2010): 23779–23789, 10.1074/jbc.M110.130658. 20501651 PMC2911289

[29] M. S. Brozzo , S. Bjelic , K. Kisko , et al., “Thermodynamic and structural description of allosterically regulated VEGFR‐2 dimerization,” Blood 119, no. 7 (2012): 1781–1788, 10.1182/blood-2011-11-390922. 22207738

[30] V. Joukov , T. Sorsa , V. Kumar , et al., “Proteolytic processing regulates receptor specificity and activity of VEGF‐C,” The EMBO Journal 16, no. 13 (1997): 3898–3911, 10.1093/emboj/16.13.3898. 9233800 PMC1170014

[31] S. Coso , Y. Zeng , K. Opeskin , and E. D. Williams , “Vascular endothelial growth factor receptor‐3 directly interacts with phosphatidylinositol 3‐kinase to regulate lymphangiogenesis,” PLoS ONE 7, no. 6 (2012): 39558, 10.1371/journal.pone.0039558. PMC3382126 22745786

[32] B. D. Manning and L. C. Cantley , “AKT/PKB signaling: Navigating downstream,” Cell 129, no. 7 (2007): 1261–1274, 10.1016/j.cell.2007.06.009. 17604717 PMC2756685

[33] S. Regot , J. J. Hughey , B. T. Bajar , S. Carrasco , and M. W. Covert , “High‐sensitivity measurements of multiple kinase activities in live single cells,” Cell 157, no. 7 (2014): 1724–1734, 10.1016/j.cell.2014.04.039. 24949979 PMC4097317

[34] M. J. Kennedy , R. M. Hughes , L. A. Peteya , J. W. Schwartz , M. D. Ehlers , and C. L. Tucker , “Rapid blue‐light–mediated induction of protein interactions in living cells,” Nature Methods 7, no. 12 (2010): 973–975, 10.1038/nmeth.1524. 21037589 PMC3059133

[35] L. J. Bugaj , A. T. Choksi , C. K. Mesuda , R. S. Kane , and D. V. Schaffer , “Optogenetic protein clustering and signaling activation in mammalian cells,” Nature Methods 10, no. 3 (2013): 249–252, 10.1038/nmeth.2360. 23377377

[36] H. Park , N. Y. Kim , S. Lee , N. Kim , J. Kim , and W. D. Heo , “Optogenetic protein clustering through fluorescent protein tagging and extension of CRY2,” Nature Communications 8, no. 1 (2017): 30, 10.1038/s41467-017-00060-2. PMC5482817 28646204

[37] L. Duan , J. Hope , Q. Ong , et al., “Understanding CRY2 interactions for optical control of intracellular signaling,” Nature Communications 8, no. 1 (2017): 547, 10.1038/s41467-017-00648-8. PMC5601944 28916751

[38] K.‐Y. Chang , D. Woo , H. Jung , et al., “Light‐inducible receptor tyrosine kinases that regulate neurotrophin signalling,” Nature Communications 5, no. 1 (2014): 4057, 10.1038/ncomms5057. 24894073

[39] A. Taslimi , J. D. Vrana , D. Chen , et al., “An optimized optogenetic clustering tool for probing protein interaction and function,” Nature Communications 5, no. 1 (2014): 4925, 10.1038/ncomms5925. PMC4170572 25233328

[40] V.‐M. Leppänen , M. Jeltsch , A. Anisimov , et al., “Structural determinants of vascular endothelial growth factor‐D receptor binding and specificity,” Blood 117, no. 5 (2011): 1507–1515, 10.1182/blood-2010-08-301549. 21148085

[41] M. Jeltsch , T. Karpanen , T. Strandin , K. Aho , H. Lankinen , and K. Alitalo , “Vascular endothelial growth factor (VEGF)/VEGF‐C mosaic molecules reveal specificity determinants and feature novel receptor binding patterns,” Journal of Biological Chemistry 281, no. 17 (2006): 12187–12195, 10.1074/jbc.M511593200. 16505489

[42] V. Joukov , V. Kumar , T. Sorsa , et al., “A recombinant mutant vascular endothelial growth factor‐C that has lost vascular endothelial growth factor receptor‐2 binding, activation, and vascular permeability activities,” Journal of Biological Chemistry 273, no. 12 (1998): 6599–6602, 10.1074/jbc.273.12.6599. 9506953

[43] T. Mäkinen , T. Veikkola , S. Mustjoki , et al., “Isolated lymphatic endothelial cells transduce growth, survival and migratory signals via the VEGF‐C/D receptor VEGFR‐3,” The EMBO Journal 20, no. 17 (2001): 4762–4773, 10.1093/emboj/20.17.4762. 11532940 PMC125596

[44] G. Jo , J. Bae , H. J. Hong , et al., “Structural insights into the clustering and activation of Tie2 receptor mediated by Tie2 agonistic antibody,” Nature Communications 12, no. 1 (2021): 6287, 10.1038/s41467-021-26620-1. PMC8560823 34725372

[45] B. da Rocha‐Azevedo , S. Lee , A. Dasgupta , et al., “Heterogeneity in VEGF Receptor‐2 Mobility and Organization on the Endothelial Cell Surface Leads to Diverse Models of Activation by VEGF,” Cell reports 32 (2020): 108187, 10.1016/j.celrep.2020.108187. 32997988 PMC7541195

[46] G. B. Whitaker , B. J. Limberg , and J. S. Rosenbaum , “Vascular Endothelial Growth Factor Receptor‐2 and Neuropilin‐1 Form a Receptor Complex That Is Responsible for the Differential Signaling Potency of VEGF165 and VEGF121,” Journal of Biological Chemistry 276, no. 27 (2001): 25520–25531, 10.1074/jbc.M102315200. 11333271

[47] Y. Chen , C. Short , A. M. Halasz , and J. S. Edwards , “The impact of high density receptor clusters on VEGF signaling,” Electronic Proceedings in Theoretical Computer Science 125 (2013): 37–52, 10.4204/EPTCS.125.3. PMC4262124 25506421

[48] T. T. Chen , A. Luque , S. Lee , S. M. Anderson , T. Segura , and M. L. Iruela‐Arispe , “Anchorage of VEGF to the extracellular matrix conveys differential signaling responses to endothelial cells,” Journal of Cell Biology 188, no. 4 (2010): 595–609, 10.1083/jcb.200906044. 20176926 PMC2828913

[49] Y. Sako , S. Minoghchi , and T. Yanagida , “Single‐molecule imaging of EGFR signalling on the surface of living cells,” Nature Cell Biology 2, no. 3 (2000): 168–172, 10.1038/35004044. 10707088

[50] X. Yu , K. D. Sharma , T. Takahashi , R. Iwamoto , and E. Mekada , “Ligand‐independent dimer formation of epidermal growth factor receptor (EGFR) is a step separable from ligand‐induced EGFR signaling,” Molecular Biology of the Cell 13, no. 7 (2002): 2547–2557, 10.1091/mbc.01-08-0411. 12134089 PMC117333

[51] N. F. Endres , R. Das , A. W. Smith , et al., “Conformational coupling across the plasma membrane in activation of the EGF receptor,” Cell 152, no. 3 (2013): 543–556, 10.1016/j.cell.2012.12.032. 23374349 PMC3718647

[52] P. Saharinen , L. Eklund , J. Miettinen , et al., “Angiopoietins assemble distinct Tie2 signalling complexes in endothelial cell–cell and cell–matrix contacts,” Nature Cell Biology 10, no. 5 (2008): 527–537, 10.1038/ncb1715. 18425119

[53] S. Fukuhara , K. Sako , T. Minami , et al., “Differential function of Tie2 at cell–cell contacts and cell–substratum contacts regulated by angiopoietin‐1,” Nature Cell Biology 10, no. 5 (2008): 513–526, 10.1038/ncb1714. 18425120

[54] J. P. Himanen , K. R. Rajashankar , M. Lackmann , C. A. Cowan , M. Henkemeyer , and D. B. Nikolov , “Crystal structure of an Eph receptor–ephrin complex,” Nature 414, no. 6866 (2001): 933–938, 10.1038/414933a. 11780069

[55] M. W. Parker , A. D. Linkugel , H. L. Goel , T. Wu , and A. M. Mercurio , “Structural basis for VEGF‐C binding to neuropilin‐2 and sequestration by a soluble splice form,” Structure 23, no. 4 (2015): 677–687, 10.1016/j.str.2015.01.018. 25752543 PMC4394031

[56] J. Garcia , H. I. Hurwitz , A. B. Sandler , et al., “Bevacizumab (Avastin®) in cancer treatment: A review of 15 years of clinical experience and future outlook,” Cancer Treatment Reviews 86 (2020): 102017, 10.1016/j.ctrv.2020.102017. 32335505

[57] P. J. Rosenfeld , D. M. Brown , J. S. Heier , et al., “Ranibizumab for neovascular age‐related macular degeneration,” New England Journal of Medicine 355, no. 14 (2006): 1419–1431, 10.1056/NEJMoa054481. 17021318

[58] G. S. Falchook , J. W. Goldman , J. Desai , et al., “A first‐in‐human phase I study of VGX‐100, a selective anti‐VEGF‐C antibody, alone and in combination with bevacizumab in patients with advanced solid tumors,” Journal of Clinical Oncology 32, no. 15 (2014): 2524–2524, 10.1200/jco.2014.32.15_suppl.2524.

[59] T. L. Jackson , J. Slakter , M. Buyse , et al., “A Randomized Controlled Trial of OPT‐302, a VEGF‐C/D Inhibitor for Neovascular Age‐Related Macular Degeneration,” Ophthalmology 130, no. 6 (2023): 588–597, 10.1016/j.ophtha.2023.02.001. 36754174

[60] M. R. Paillasse , M. Esquerré , F. A. Bertrand , et al., “Targeting Tumor Angiogenesis with the Selective VEGFR‐3 Inhibitor EVT801 in Combination with Cancer Immunotherapy,” Cancer Research Communications 2, no. 11 (2022): 1504–1519, 10.1158/2767-9764.CRC-22-0151. 36970050 PMC10035370

[61] M. Jeltsch , S. K. Jha , D. Tvorogov , et al., “CCBE1 Enhances Lymphangiogenesis via A Disintegrin and Metalloprotease With Thrombospondin Motifs‐3–Mediated Vascular Endothelial Growth Factor‐C Activation,” Circulation 129, no. 19 (2014): 1962–1971, 10.1161/CIRCULATIONAHA.113.002779. 24552833

[62] H. M. Jopling , G. J. Howell , N. Gamper , and S. Ponnambalam , “The VEGFR2 receptor tyrosine kinase undergoes constitutive endosome‐to‐plasma membrane recycling,” Biochemical and Biophysical Research Communications 410, no. 2 (2011): 170–176, 10.1016/j.bbrc.2011.04.093. 21539813 PMC6103438

[63] A. Punjani , J. L. Rubinstein , D. J. Fleet , and M. A. Brubaker , “cryoSPARC: Algorithms for rapid unsupervised cryo‐EM structure determination,” Nature Methods 14, no. 3 (2017): 290–296, 10.1038/nmeth.4169. 28165473

[64] S. Q. Zheng , E. Palovcak , J. P. Armache , K. A. Verba , Y. Cheng , and D. A. Agard , “MotionCor2: Anisotropic correction of beam‐induced motion for improved cryo‐electron microscopy,” Nature Methods 14, no. 4 (2017): 331–332, 10.1038/nmeth.4193. 28250466 PMC5494038

[65] D. Tegunov and P. Cramer , “Real‐time cryo‐electron microscopy data preprocessing with Warp,” Nature Methods 16, no. 11 (2019): 1146–1152, 10.1038/s41592-019-0580-y. 31591575 PMC6858868

[66] S. H. Scheres and S. Chen , “Prevention of overfitting in cryo‐EM structure determination,” Nature Methods 9, no. 9 (2012): 853–854, 10.1038/nmeth.2115. 22842542 PMC4912033

[67] P. B. Rosenthal and R. Henderson , “Optimal determination of particle orientation, absolute hand, and contrast loss in single‐particle electron cryomicroscopy,” Journal of Molecular Biology 333, no. 4 (2003): 721–745, 10.1016/j.jmb.2003.07.013. 14568533

[68] G. Cardone , J. B. Heymann , and A. C. Steven , “One number does not fit all: Mapping local variations in resolution in cryo‐EM reconstructions,” Journal of Structural Biology 184, no. 2 (2013): 226–236, 10.1016/j.jsb.2013.08.002. 23954653 PMC3837392

[69] R. Sanchez‐Garcia , J. Gomez‐Blanco , A. Cuervo , J. M. Carazo , C. O. S. Sorzano , and J. Vargas , “DeepEMhancer: A deep learning solution for cryo‐EM volume post‐processing,” Communications Biology 4, no. 1 (2021): 874, 10.1038/s42003-021-02399-1. 34267316 PMC8282847

[70] J. Abramson , J. Adler , J. Dunger , et al., “Accurate structure prediction of biomolecular interactions with AlphaFold 3,” Nature 630, no. 8016 (2024): 493–500, 10.1038/s41586-024-07487-w. 38718835 PMC11168924

[71] P. Emsley and K. Cowtan , “Coot: Model‐building tools for molecular graphics,” Acta Crystallographica Section D Biological Crystallography 60, no. 12 (2004): 2126–2132, 10.1107/S0907444904019158. 15572765

[72] P. D. Adams , P. V. Afonine , G. Bunkóczi , et al., “PHENIX: A comprehensive Python‐based system for macromolecular structure solution,” Acta Crystallographica Section D Biological Crystallography 66, no. 2 (2010): 213–221, 10.1107/S0907444909052925. 20124702 PMC2815670

[73] E. F. Pettersen , T. D. Goddard , C. C. Huang , et al., “UCSF ChimeraX: Structure visualization for researchers, educators, and developers,” Protein Science 30, no. 1 (2021): 70–82, 10.1002/pro.3943. 32881101 PMC7737788

