# Supplementary material for: Structural Basis of Lymphangiogenic Receptor VEGFR‐3 Activation Mediated by Distinctive Clustering of the Ligand–Receptor Complex
Source: Adv Sci (Weinh). 2026 Sep 9:e77728. Online ahead of print. doi: 10.1002/advs.77728 (PMC13558912; doi:10.1002/advs.77728)
Supplement: Supplementary file 1 [file ADVS-9999-e77728-s001.pdf]

Supplementary Tables

Table S1. Cryo-EM data, collection, refinement, and validation statistics

**Data collection and processing**	*Cis*-clustered VEGFR-3/VEGF-C complex	*Trans*-like-clustered VEGFR-3/VEGF-C complex
Magnification	105,000	105,000
Voltage (kV)	300	300
Electron exposure (e^-/Å^2)	67.3	64.8
Defocus range (µm)	-0.7 ~ -1.9	-0.7 ~ -2.2
Pixel size (Å)	0.848	0.849
Symmetry imposed	C1	C1
Initial particle images (no.)	3,395,573	2,217,730
Final particle images (no.)	580,022	486,815
Map resolution (Å)	3.34	3.23
FSC threshold	0.143	0.143
**Refinement**		
Initial model used (PDB code)	4BSK,4BSJ, AlphaFold Prediction	4BSK,4BSJ, AlphaFold Prediction
Map resolution (Å)	3.3	3.3
FSC threshold	0.143	0.143
Map sharpening B factor (Å^2)	0	0
**Model composition**		
Nonhydrogen atoms	15,441	11,865
Protein residues	1947	1500
Ligands	0	0
**B factors (Å^2)**		
Protein	95.6	95.7
Ligand	0	0
**R.m.s. deviations**		
Bond lengths (Å)	0.003	0.005
Bond angles (°)	0.688	0.716
**Validation**		
MolProbity score	2.20	2.12
Clashscore	5.00	3.93
Poor rotamers (%)	5.56	5.50
**Ramachandran plot**		
Favored (%)	94.6	94.4
Allowed (%)	5.4	5.6
Disallowed (%)	0.05	0.00
PDB ID	21ES	21EI

Table S2. Interacting residues for the canonical 2:2 VEGFR-3/VEGF-C complex and high-order assemblies

- Interaction residues between VEGF-C and VEGFR-3

Region	VEGFR-3^{chain}	VEGF-C^{chain}	Interaction	Distance (Å)
A (D2)	P143^{B}	P196^{D}	hydrophobic	3.9
	L147^{B}	V190^{D}	hydrophobic	4.3
		L192^{D}	hydrophobic	3.7
		I122^{C}	hydrophobic	3.7,4.9
	L202^{B}	D123^{C}	hydrogen	3.2
		W126^{C}	hydrophobic	4.2
	Y203^{B}	P154^{D}	hydrophobic	4.1
	P219^{B}	L119^{C}	hydrophobic	4.2
		F186^{D}	hydrophobic	3.8
	L221^{B}	I188^{D}	hydrophobic	4.5
		P196^{D}	hydrophobic	3.6
	H223^{B}	F151^{D}	hydrophobic	4.0
	V222^{B}	K153^{D}	hydrogen	2.8
B (D3)	N259^{B}	S168^{C}	hydrogen	2.9
	V262^{B}	T148^{D}	hydrogen	3.2
	T263^{B}	T148^{D}	hydrogen	3.3
	R282^{B}	N149^{D}	hydrogen	3.0
	Q285^{B}	E169^{C}	ionic, hydrogen	3.0, 3.4
	T289^{B}	K214^{C}	hydrogen	3.3
	**VEGFR-3^{chain}**	**VEGFR-3^{chain}**	**Interaction**	
C (D5)	K516^{A}	E428^{B}	hydrogen	
	H425^{A}	E428^{B}	ionic	
	T446^{A}	S430^{B}	hydrogen	
	E509^{A}	S430^{B}	hydrogen	

- *Cis*-clustering interaction residues

Region	VEGFR-3^{chian} (D2)	VEGF-C^{chain}	Interaction	Distance (Å)
Upper	Q181^{E,B}	S208^{C,H}	hydrogen	3.5
	Q181^{E,B}	S177^{C,H}	hydrogen	2.9
	E182^{E,B}	T176^{C,H}	hydrogen	3.0
**Region**	**VEGFR-3^{chain} (D5)**	**VEGFR-3^{chain} (D5)**	**Interaction**	**Distance (Å)**
Lower	W463^{B}	W463^{E}	hydrophobic	3.5
	P465^{B}	P465^{E}	hydrophobic	3.6,4.4
	M531^{B}	M531^{E}	hydrophobic	4.4

- *Trans*-like-clustering interaction residues

Region	VEGFR3^{chain} (D3)	VEGFR3^{chain} (D1)	Interaction	Distance (Å)
D3-D1		S26^{E}	hydrogen	3.2
	Y230^{B}	H55^{E}	hydrophobic	4.4
	I232^{B}	I116^{E}	hydrophobic	3.7
	Q317^{B}	Y115^{E}	hydrophobic	4.0,3.6
	F319^{B}	K117^{E}	hydrogen	3.2

The residues participating in the main chain interaction are underlined.

Table S3. Buried area of interaction interface between VEGF-C and VEGFR D2-D3 domain

Buried area(Å^2)		VEGF-C^{chainC}	VEGF-C^{chainD}	Total
**VEGFR-2**	**D2**	370.88	272.31	**643.19**
	**D3**	186.79	319.28	**506.07**
**Total**		**557.67**	**591.59**	**1149.26**
**VEGFR-3**	**D2**	304.81	413.46	**736.27**
	**D3**	235.96	281.51	**517.47**
**Total**		**540.77**	**694.97**	**1235.74**

Table S4. Primer sequences used for cloning (species: human)

Primer	Sequence (5'-3')
VEGF-C_F	TTC TCT CCA CAG AAG CTT GCC GCC ATG GTG TCC TAC T
VEGF-C_R	ATG ATG ATG ATG GGA TCC CAG TTT AGA CAT GCA TCG G
VEGF-C (C137A)_F	TGC ATG CCA CGG GAG GTG GCT ATA GAT GTG GGG AAG GAG
VEGF-C (C137A)_R	CTC CTT CCC CAC ATC TAT AGC CAC CTC CCG TGG CAT GCA
VEGFR-3 (WT)-3C_F	TCT AGT TCA GGT GGA TCC ATG CAG CGG GGG CGC CGC GCT GTG CC
VEGFR-3 (WT)-3C_R	CGC GGA ACC AGT GCG GCC GCT GGT CCC TGA AAC AGC ACC TCA AGC TTA AGC TCC TCG GAG CCT TCC ACG GCC ACG C
VEGFR-3 (W463E/T464E/P465E)-3C_F1	TCT AGT TCA GGT GGA TCC ATG CAG CGG GGG CGC CGC GCT GTG CC
VEGFR-3 (W463E/T464E/P465E)-3C_R1	GAC TAC GCT GGG CAA ACA TCT TGC ACT CCT CCT CGG GCC GCC AGT GCC ACT GGA TGC TG
VEGFR-3 (W463E/T464E/P465E)-3C_F2	CAG CAT CCA GTG GCA CTG GCG GCC CGA GGA GGA GTG CAA GAT GTT TGC CCA GCG TAG TC
VEGFR-3 (W463E/T464E/P465E)-3C_R2	CAG CAC CTC AAG CTT AAG ATC CTC GGA GCC TTC CAC GGC CAC GC
VEGFR-3 (H55A/Y115A/Y230A)-3C_F1	TCT AGT TCA GGT GGA TCC ATG CAG CGG GGG CGC CGC GCT GTG CC
VEGFR-3 (H55A/Y115A/Y230A)-3C_R1	CCA CTC GAG GGG GGC CTG TCC CCT GCA G
VEGFR-3 (H55A/Y115A/Y230A)-3C_F2	CTG CAG GGG ACA GGC CCC CTC GAG TGG
VEGFR-3 (H55A/Y115A/Y230A)-3C_R2	CTC GAT GCG TGC CTT GAT GGC CTT GTA GTA GCA GAC GTA G
VEGFR-3 (H55A/Y115A/Y230A)-3C_F3	CTA CGT CTG CTA CTA CAA GGC CAT CAA GGC ACG CAT CGA G
VEGFR-3 (H55A/Y115A/Y230A)-3C_R3	GCA ACA GCT GGA TGT CAG CGA GCT CGT TGC CTG TGA
VEGFR-3 (H55A/Y115A/Y230A)-3C_F4	TCA CAG GCA ACG AGC TCG CTG ACA TCC AGC TGT TGC
VEGFR-3 (H55A/Y115A/Y230A)-3C_R4	CAG CAC CTC AAG CTT AAG ATC CTC GGA GCC TTC CAC GGC CAC GC
VEGFR-3 (H55A/Y115A/Y230A/W463E/ T464E/P465E)-3C_F1	TCT AGT TCA GGT GGA TCC ATG CAG CGG GGG CGC CGC GCT GTG CCA
VEGFR-3 (H55A/Y115A/Y230A/W463E/ T464E/P465E)-3C_R1	GAC TAC GCT GGG CAA ACA TCT TGC ACT CCT CCT CGG GCC GCC AGT GCC ACT GGA TGC TG
VEGFR-3 (H55A/Y115A/Y230A/W463E/ T464E/P465E)-3C_F2	CAG CAT CCA GTG GCA CTG GCG GCC CGA GGA GGA GTG CAA GAT GTT TGC CCA GCG TAG TC
VEGFR-3 (H55A/Y115A/Y230A/W463E/ T464E/P465E)-3C_R2	CAG CAC CTC AAG CTT AAG ATC CTC GGA GCC TTC CAC GGC CAC GC
VEGFR-3 (WT)-EGFP_F	ACC GTC AGA TCC GCT AGC ATG CAG CGG GGG CGC C
VEGFR-3 (WT)-EGFP_R	GTG GCG ACC GGT GGA TCC CCC GAT CCT CCT CCG CCA GAA CCT CCC CCT CCG TAG CTG TTG TCT GTG AAG AAA GTC ACG
VEGFR-3 (W463E/T464E/P465E)-EGFP_F1	ACC GTC AGA TCC GCT AGC ATG CAG CGG GGG CGC C
VEGFR-3 (W463E/T464E/P465E)-EGFP_R1	GAC TAC GCT GGG CAA ACA TCT TGC ACT CCT CCT CGG GCC GCC AGT GCC ACT GGA TGC TG
VEGFR-3 (W463E/T464E/P465E)-EGFP_F2	CAG CAT CCA GTG GCA CTG GCG GCC CGA GGA GGA GTG CAA GAT GTT TGC CCA GCG TAG TC
VEGFR-3 (W463E/T464E/P465E)-EGFP_R2	GTG GCG ACC GGT GGA TCC CCC GAT CCT CCT CCG CCA GAA CCT CCC CCT CCG TAG CTG TTG TCT GTG AAG AAA GTC ACG
VEGFR-3 (WT)-CRY2PHR-EGFP_F	ACC GTC AGA TCC GCT AGC ATG CAG CGG GGG CGC C
VEGFR-3 (WT)-CRY2PHR-EGFP_R	CTG CAG AAT TCG AAG CTT GCG TAG CTG TTG TCT GTG AAG AAA GTC ACG
VEGFR-3 (W463E/T464E/P465E) -CRY2PHR-EGFP_F1	ACC GTC AGA TCC GCT AGC ATG CAG CGG GGG CGC CGC GCT
VEGFR-3 (W463E/T464E/P465E) -CRY2PHR-EGFP_R1	GAC TAC GCT GGG CAA ACA TCT TGC ACT CCT CCT CGG GCC GCC AGT GCC ACT GGA TGC TG
VEGFR-3 (W463E/T464E/P465E) -CRY2PHR-EGFP_F2	CAG CAT CCA GTG GCA CTG GCG GCC CGA GGA GGA GTG CAA GAT GTT TGC CCA GCG TAG TC
VEGFR-3 (W463E/T464E/P465E) -CRY2PHR-EGFP_R2	CTG CAG AAT TCG AAG CTT GCG TAG CTG TTG TCT GTG AAG AAA GTC ACG CGG GCA GAC G
