# Supplementary material for: Structural Basis of Lymphangiogenic Receptor VEGFR‐3 Activation Mediated by Distinctive Clustering of the Ligand–Receptor Complex
Source: Adv Sci (Weinh). 2026 Sep 9:e77728. Online ahead of print. doi: 10.1002/advs.77728 (PMC13558912; doi:10.1002/advs.77728)
Supplement: Supplementary file 2 [file ADVS-9999-e77728-s002.pdf]

Supporting Information

Structural basis of lymphangiogenic receptor VEGFR-3 activation mediated by distinctive clustering of the ligand–receptor complex

Ryeongeun Cho[1,2,7], Jinsook Ahn[3,7], Jimin Yang[4], Dong Sun Lee[3], Gahi Hong[4,6], Sangkyu Lee[5,#], and Ho Min Kim[1,3,#]

[1]InnoCORE AI-CRED Institute, Korea Advanced Institute of Science and Technology (KAIST), Daejeon, 34141, Republic of Korea

[2]Graduate School of Medical Science and Engineering, Korea Advanced Institute of Science and Technology (KAIST), Daejeon, 34141, Republic of Korea

[3]Department of Biological Sciences, Korea Advanced Institute of Science and Technology (KAIST), Daejeon, 34141, Republic of Korea

[4]Department of Molecular Biology, Jeonbuk National University, Jeonju, 54896, Republic of Korea

[5]Center for Memory and Glioscience, Institute for Basic Science (IBS), Daejeon, 34126, Republic of Korea

[6]Research Institute for Materials and Energy Sciences, Jeonbuk National University, Jeonju, 54896, Republic of Korea

[7]These authors contributed equally to this work

[#]Corresponding authors: H. M. Kim (hm_kim@kaist.ac.kr), or S. Lee (sklee@ibs.re.kr)

This PDF file includes

Supplementary Fig. 1-8

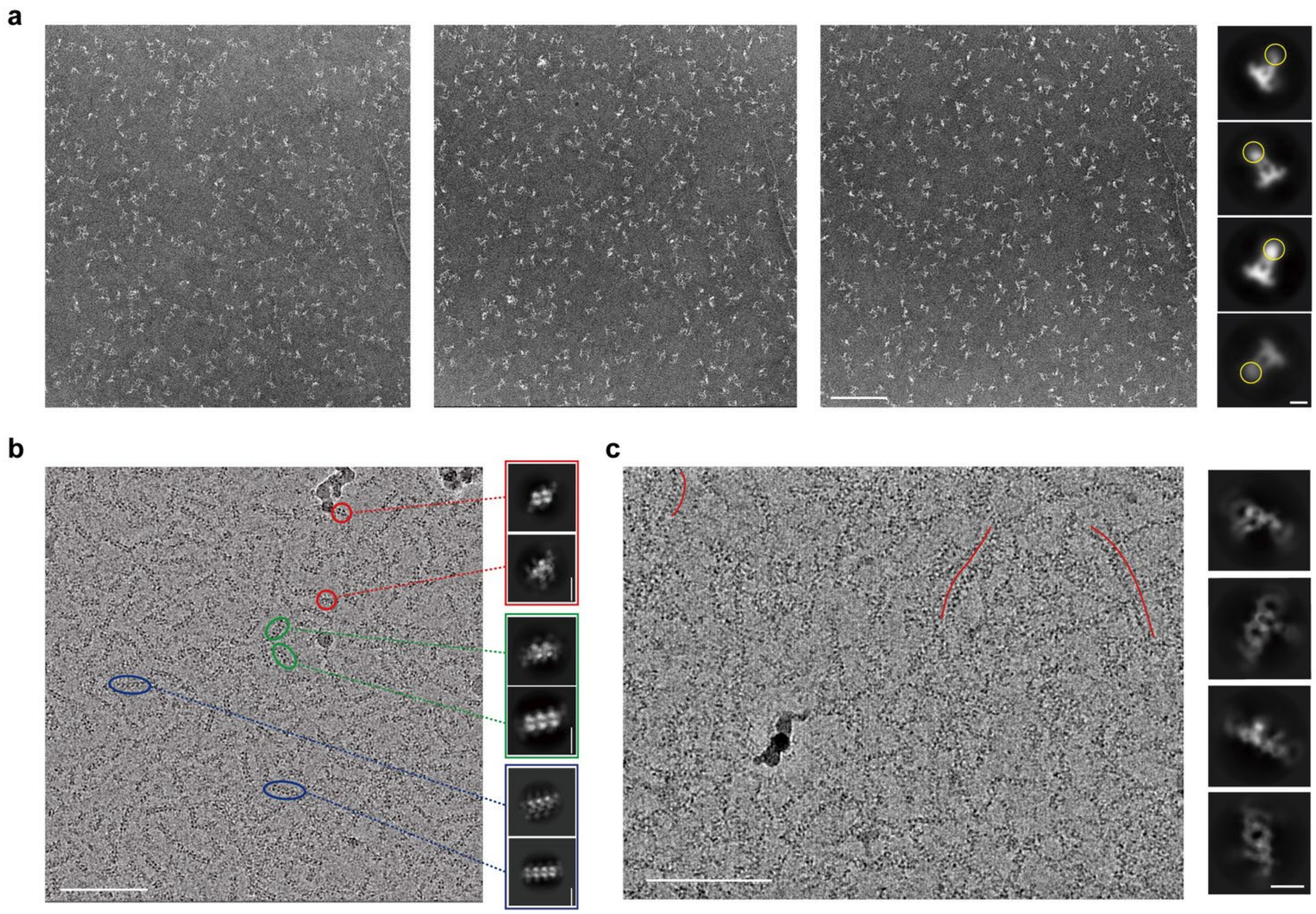

Supplementary Fig1. Raw EM micrographs and 2D class averages of the VEGFR-3/VEGF-C complex and its clustering states.

a Representative negative-stain TEM micrographs (left) and 2D class averages (right) of the [VEGFR-3/VEGF-C]$_2$ complex. Scale bars, 100 nm (micrographs), 100 Å (2D class averages). The flexible membrane-proximal region is indicated by yellow circles within the 2D class averages.

b A representative cryo-EM micrograph showing *cis*-clustered assemblies of the VEGFR-3/VEGF-C complex. Corresponding 2D class averages for the encircled particles are shown on the right. The 2-, 3-, and 4-unit clusters are indicated by red, green, and blue circles, respectively. Scale bars: 100 nm (micrograph) and 100 Å (2D class averages).

c A representative cryo-EM micrograph showing *trans*-like-clustered assemblies of the VEGFR-3/VEGF-C complex. The assemblies are marked with red lines, and representative 2D class averages are shown on the right. Scale bars: 100 nm (micrograph) and 100 Å (2D class averages).

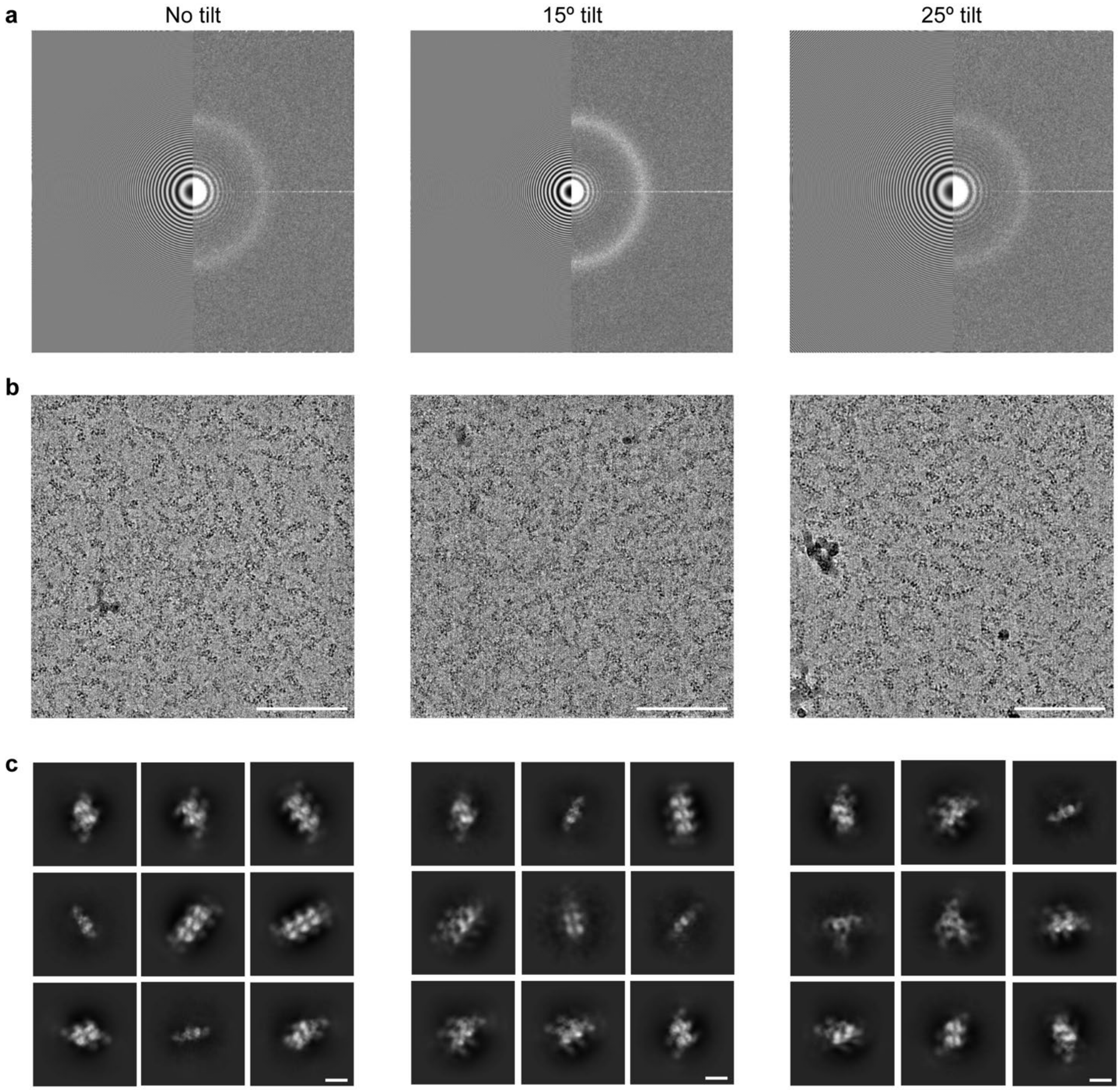

(Continued)

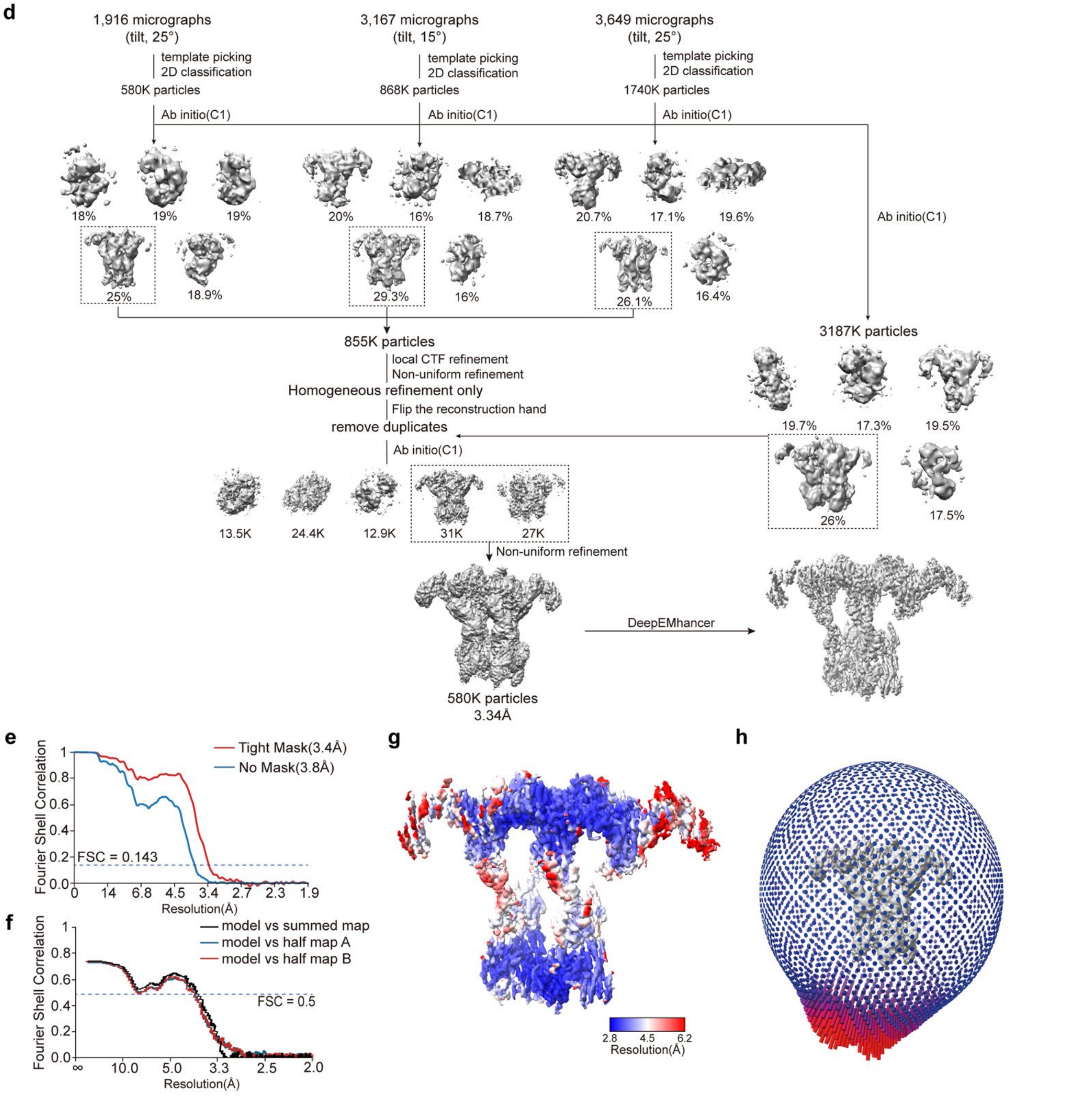

Supplementary Fig2. Cryo-EM analysis of the *cis*-clustered VEGFR-3/VEGF-C complex

a–c Representative Fourier transforms **(a)**, cryo-EM micrographs **(b)** and representative 2D class averages **(c)** of *cis*-clustered VEGFR-3/VEGF-C complexes acquired without tilt (left), and with tilt at 15° (center), and at 25° (right). Scale bars, 100 nm (micrographs), 100 Å (2D class averages).

d Data processing workflow employed for the *cis*-clustered VEGFR-3/VEGF-C complex.

e Fourier shell correlation (FSC) curves between two independently refined half-maps, with a resolution cutoff of FSC = 0.143.

f FSC curves for cross-validating the final atomic model against the summed and half-maps, with a resolution cutoff of FSC = 0.5.

g The final cryo-EM density map of the *cis*-clustered VEGFR-3/VEGF-C complex with colors indicating the local resolutions.

h Euler angle distribution of all particles used for the final 3D reconstruction. The height and color gradient (ranging from blue to red) of the cylinder bars indicate the relative number of particles present in each orientation.

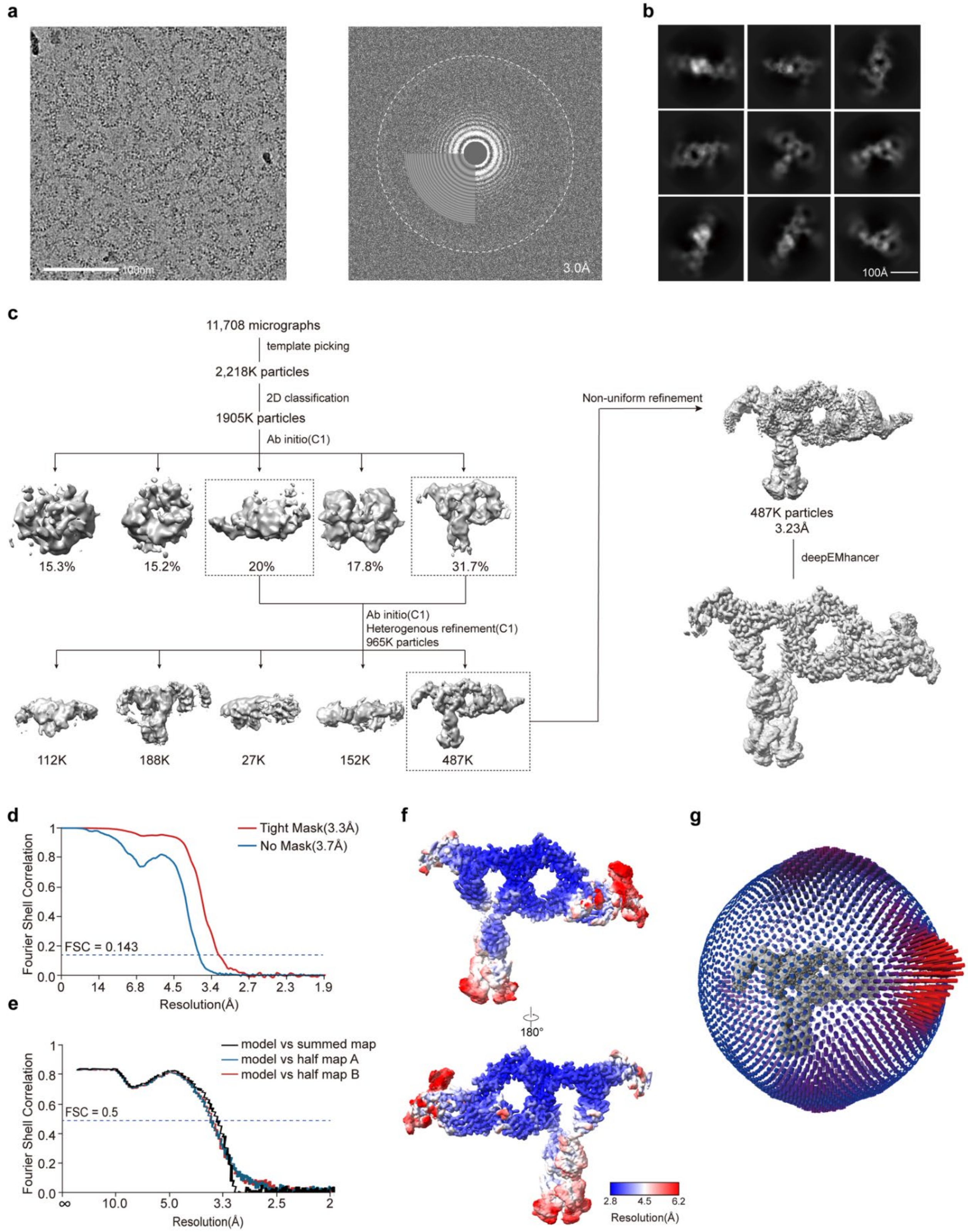

Supplementary Fig3. Cryo-EM analysis of the *trans*-like-clustered VEGFR-3/VEGF-C complex

a A representative cryo-EM micrograph of the *trans*-like-clustered VEGFR-3/VEGF-C complex (left) and its Fourier transform (right). Scale bars: 100 nm.

b Representative 2D class averages of *trans*-like-clustered VEGFR-3/VEGF-C complex. Scale bar: 100Å.

c The data processing workflow employed for the *trans*-like-clustered VEGFR-3/VEGF-C complex.

d Fourier shell correlation (FSC) curves between the two independently refined half-maps, with a resolution cutoff of FSC = 0.143.

e FSC curves for cross-validating the final atomic model against the summed and half-maps, with a resolution cutoff of FSC = 0.5.

f The final cryo-EM density map of the *trans*-like-clustered VEGFR-3/VEGF-C complex with colors indicating the local resolutions.

g Euler angle distribution of all particles used for the final 3D reconstruction. The height and color gradient (ranging from blue to red) of the cylinder bars indicate the relative number of particles present in each orientation.

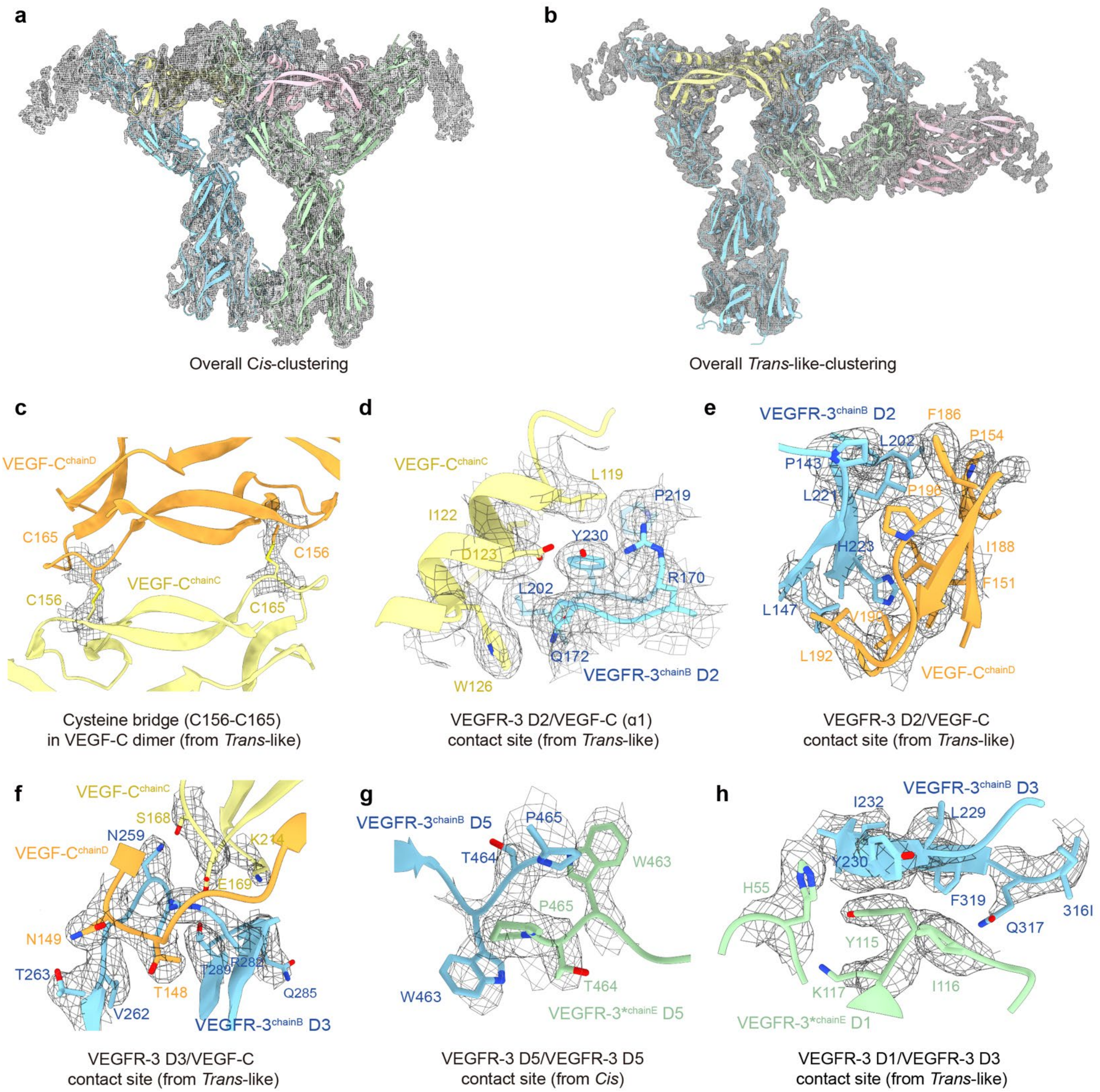

Supplementary Fig4. Cryo-EM density map for the *cis*- and *trans*-like-clustered VEGFR-3/VEGF-C complexes

a and b A Cryo-EM density map (mesh, contour level : 0.06 for *cis*-clustering, 0.105 for *trans*-like-clustering) and atomic model (cartoon) for the overall *cis*- and *trans*-like-clustered VEGFR-3/VEGF-C complexes. The color scheme is identical to the one used in **Figs. 1d** and **1e**.

c A close-up view of the intermolecular disulfide bridges (C156–C165) within the VEGF-C dimer, presented with the corresponding cryo-EM density map (mesh, contour level : 0.104).

d-f Cryo-EM density maps [mesh, contour level : 0.0932 **(d)**, 0.175 **(e, f)**] and atomic model (cartoon and stick), showing the interface between VEGF-C^{chainC} N-terminal $\alpha1$ helix and VEGFR-3^{chainB} D2 **(d),** VEGF-C^{chainD} and VEGFR-3^{chainB} D2 **(e),** and VEGF-C^{chainC} L1 and VEGFR-3^{chainB} D3 **(f)**.

g and h Cryo-EM density maps [mesh, contour level : 0.0941 **(g)**, 0.173 **(h)**] and atomic model (cartoon and stick) of the clustering interface between VEGFR-3^{chainB} D5 and VEGFR-3*^{chainE} D5 for *cis*-clustered complex **(g)**, and between VEGFR-3^{chainB} D3 and VEGFR-3*^{chainE} D1 for the *trans*-like-clustered complex **(h)**.

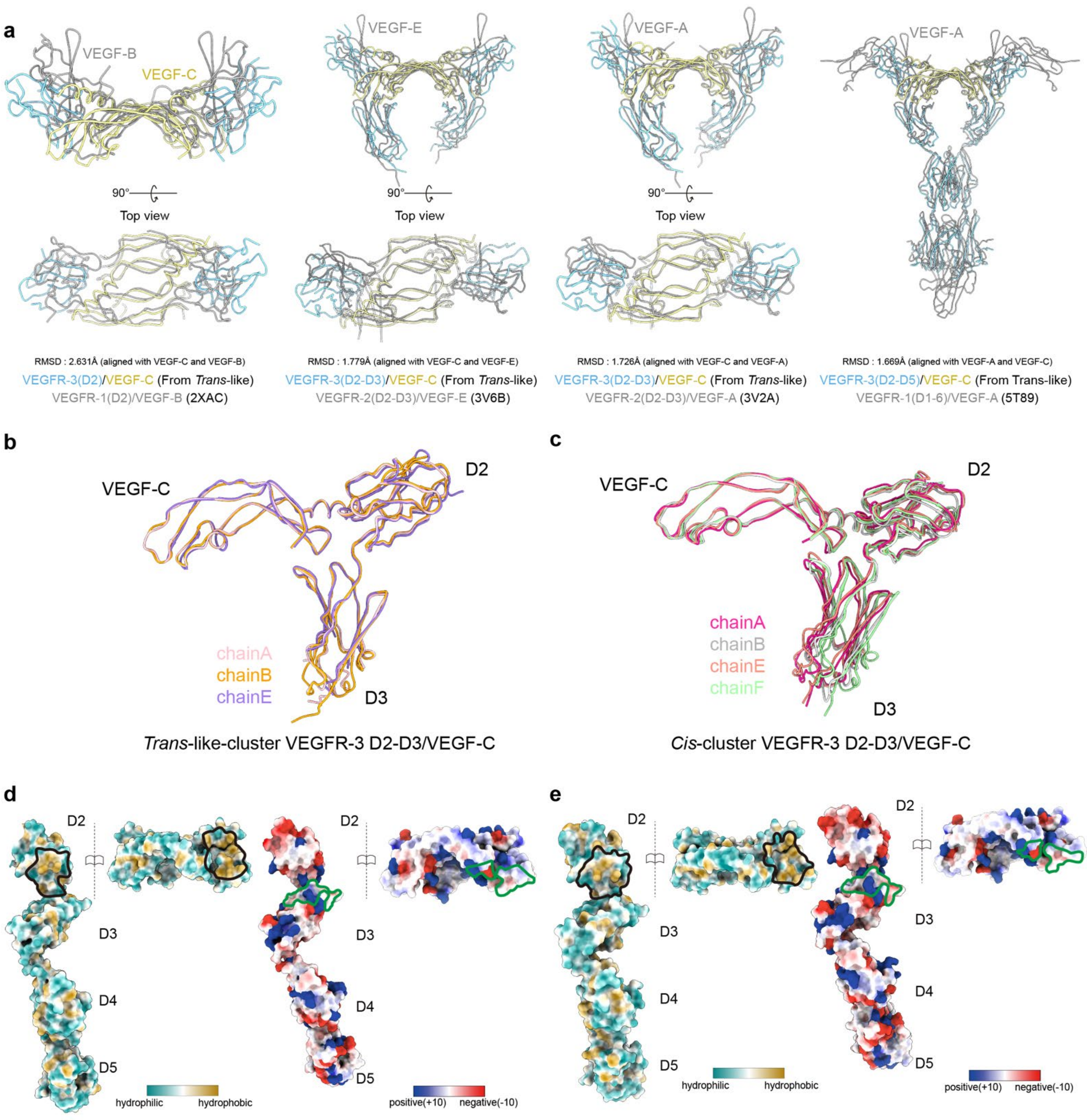

Supplementary Fig5. Structural comparison of the VEGFRs/VEGFs complexes and interaction surfaces

a Structural comparison of the indicated canonical 2:2 VEGFR-3 ECD/VEGF-C complex (from *trans*-like-clustered assembly, colored) with other VEGFR/VEGF family complexes (gray). From left to right, superposition with [VEGFR-1 D2/VEGF-B]$_2$ complex (PDB: 2XAC)[28], [VEGFR-2 D2-D3/VEGF-E]$_2$ complex (3V6B)[29], [VEGFR-2 D2-D3/VEGF-A]$_2$ complex (PDB : 3V2A)[29], [VEGFR-1 D1-D6/VEGF-A]$_2$ complex (PDB : 5T89)[13]. All RMSD values are calculated based on the aligned VEGF molecules. All structures are represented as ribbons.

b Structural comparison of three VEGFR-3 D2–D3/VEGF-C protomers from the *trans*-like-clustered assembly.

c Structural comparison of four VEGFR-3 D2–D3/VEGF-C protomers from the *cis*-clustered assembly.

a-c Structures were aligned with one VEGF-C protomer of VEGF-C dimer.

d and e Hydrophobic surface (left) and electrostatic potential (right) of the VEGFR-3 D2-D5/VEGF-C complex shown in open-book representations (from *trans*-like-clustered **(d)** and from *cis*-clustered **(e)** assembly). Hydrophilic (teal) to hydrophobic (brown) regions are colored accordingly. Key hydrophobic interaction surfaces are outlined in black (left). Charge distribution is colored: positive (blue) to negative (red). Key electrostatic interaction surfaces (interactions between VEGFR-3 D3 and VEGF-C) are outlined in green (right).

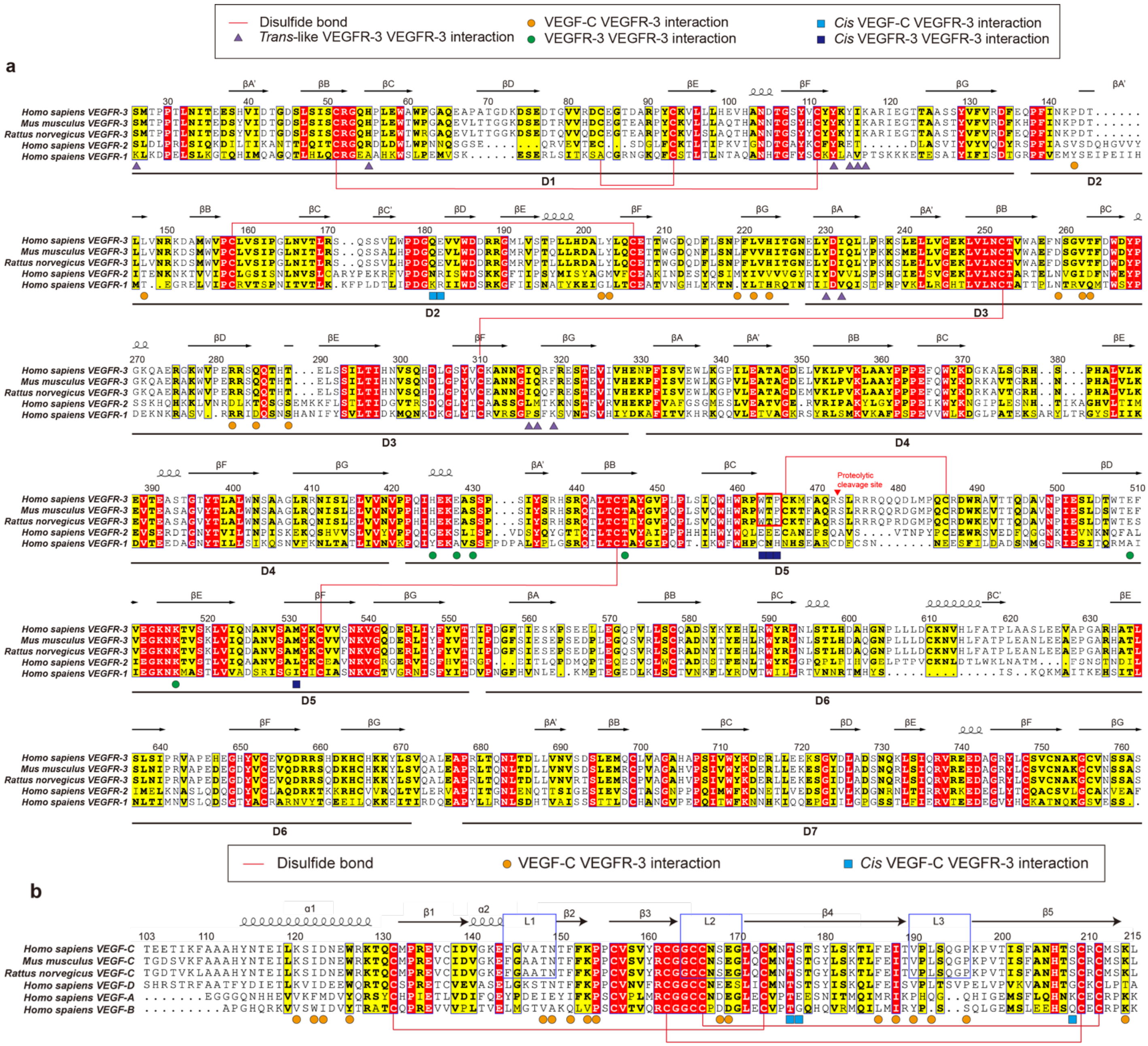

Supplementary Fig6. Multiple sequence alignment of VEGFR and VEGF family members

a Amino acid sequence alignment of human (*Homo sapiens*, UniProt: P35916), mouse (*Mus musculus*, UniProt: P35917), and rat (*Rattus norvegicus*, UniProt: Q91ZT1) VEGFR-3 ECD with human VEGFR-2 (*Homo sapiens*, UniProt: P35968), and human VEGFR-1 (*Homo sapiens*, UniProt: P17948). Domains (D1-D7) are marked below the alignment. The WTP motif in *cis*-clustering is highlighted with a red box.

b Amino acid sequence alignment of human (*Homo sapiens*, UniProt: P49767), mouse (*Mus musculus*, UniProt: P97953), and rat (*Rattus norvegicus*, UniProt: Q35757) VEGF-C with human VEGF-A (*Homo sapiens*, UniProt: P15692), human VEGF-D (*Homo sapiens* ,UniProt: O43915) and human VEGF-B (*Homo sapiens*, UniProt: P49765). The three key loops involved in the interactions (L1, L2 and L3) are indicated as blue boxes.

a and b Secondary structure elements (arrows: β-sheets; coils: α-helices) are shown above the alignments. Key structural features are indicated: Disulfide bonds (red lines), $[VEGFR\text{-}3/VEGF\text{-}C]_2$ complex interactions (orange circles, VEGF-

C/VEGFR-3^{chainB}; and green circles, VEGFR-3^{chainA}/VEGFR-3^{chainB}), *cis*-clustering interactions (cyan square, VEGF-C^{chainC}/VEGFR-3*^{chainE} or VEGF-C^{chainH}/VEGFR-3^{chainB}; blue square, VEGFR-3^{chainB}/VEGFR-3*^{chainE}), and *trans*-like-clustering interactions (purple triangles). Fully conserved residues are boxed in red; residues with >70% similarity based on physicochemical properties are shaded yellow. The sequence alignments were generated using Clustal Omega[67] (https://www.ebi.ac.uk/Tools/msa/clustalo/) and visualized with EsPript 3.0[68] (https://espript.ibcp.fr/ESPript/ESPript/).

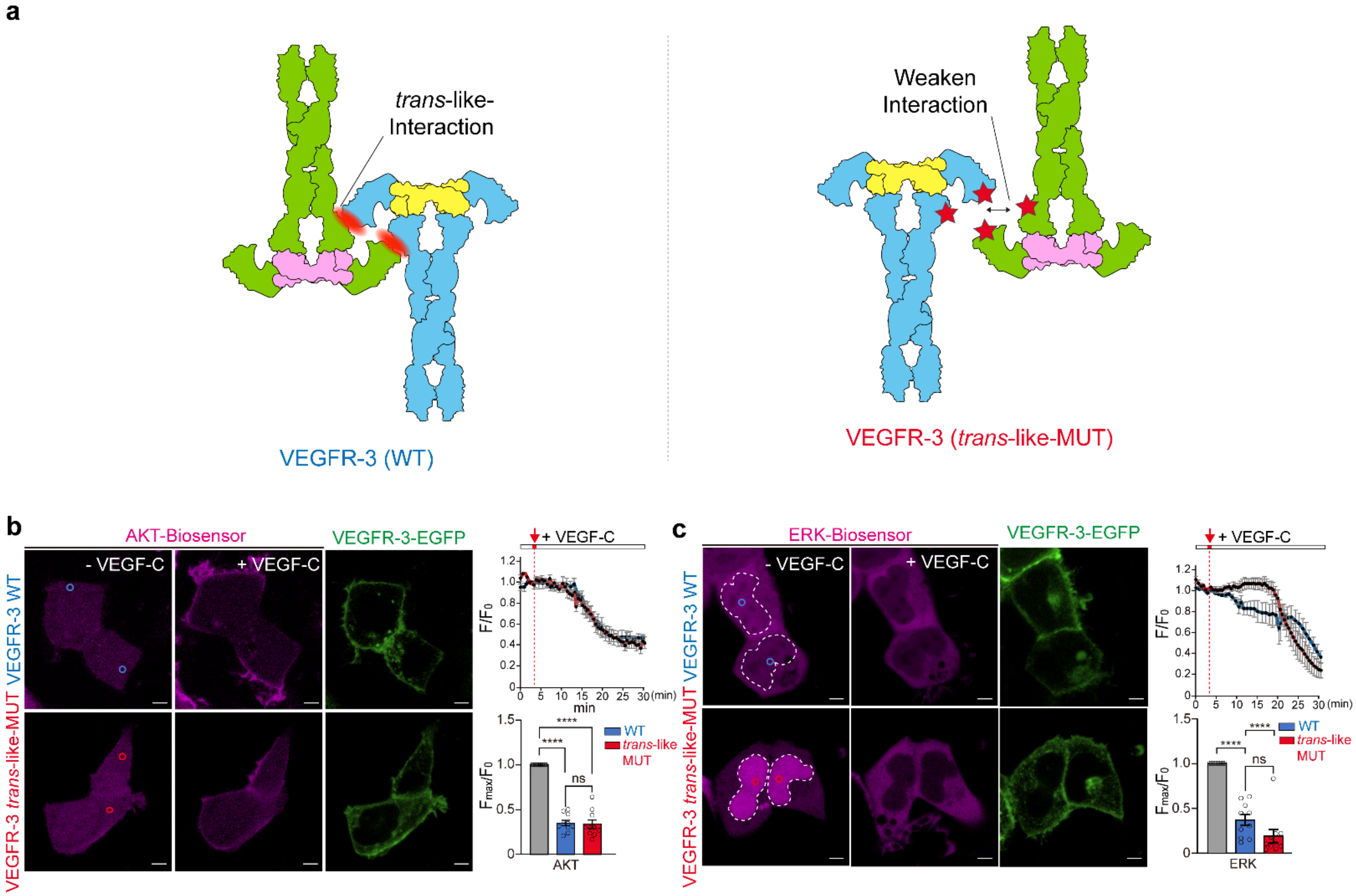

Supplementary Fig7. Functional analysis of the VEGFR-3 *trans*-like-clustering mutant (*trans*-like-MUT) in response to VEGF-C

a Schematic diagram of the VEGFR-3 wild-type (WT) and *trans*-like-clustering mutant (*trans*-like-MUT) constructs. The *trans*-like-interaction region in D1 and D3 (H55, Y115, and Y230) is highlighted with red stars. In the *trans-like*-MUT, these key residues were mutated to alanine (H55A, Y115A, and Y230A) to weaken the interaction.

b and c Representative live-cell images showing AKT (b) and ERK (c) activation in VEGFR-3 WT or trans-like-MUT-expressing cells 25 mins after VEGF-C treatment (100 ng/ml) (left). Dashed lines indicate the nucleus, and colored circles mark regions of interest (ROI) for the quantification of fluorescence intensity (F) of the AKT and ERK biosensors. Scale bars: 5 µm.

Time-lapse quantification of AKT **(b)** and ERK biosensors **(c)** at ROI after VEGF-C treatment (right, top). Fluorescence intensity (F) was normalized to the baseline F_0 at t = 0. VEGF-C (100 ng/ml) was added at 2–3 min (red dashed line and arrow). Blue and red lines represent F changes in VEGFR-3 WT and *trans*-like-MUT-expressing cells, respectively. Data are the mean ± SEM (WT, n = 10; and *cis*-MUT, n = 10). Bar graphs represent the F_{max}/F_0 ratio (right, bottom), where t = max is the time point with maximal intensity change. The gray bar indicates F_0/F_0. Each dot represents data from an individual cell, and data are represented as mean ± SEM (WT, n = 10; and *trans*-like-MUT, n = 10). Statistical significance was calculated using one-way ANOVA with Tukey's multiple comparisons (ns, not significant; ****$p < 0.0001$, ***$p < 0.001$).

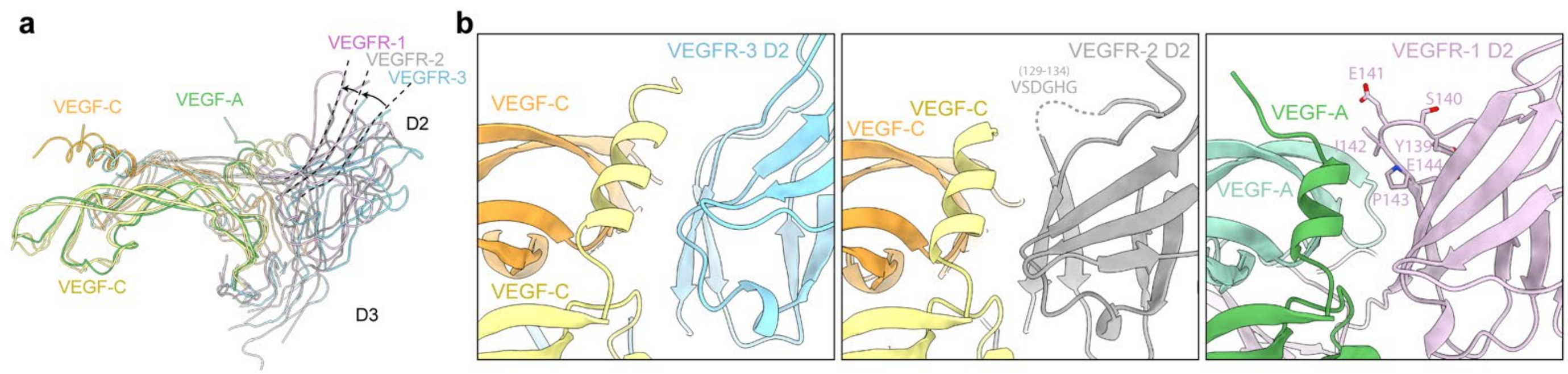

Supplementary Fig8. Structural basis for the inability of VEGF-C to bind to VEGFR-1

a Structural comparison of the VEGFR-3 D2–D3/VEGF-C (cyan), VEGFR-2 D2–D3/VEGF-C (gray, PDB : 2X1W)[24] and VEGFR-1 D2–D3/VEGF-A (purple, PDB : 5T89)[13], aligned with VEGF-C and VEGF-A protomers.

b Close-up views of the interacting interface between VEGF and VEGFR-D2 (VEGFR-3/VEGF-C, left; VEGFR-2/VEGF-C, middle; and VEGFR-1/VEGF-A, right). The D1–D2 connecting loop of the VEGFR-2 is indicated with black dashed lines. The D1–D2 connecting loop of VEGFR-1 (“YSEIPE”) is shown as sticks in the right panel.
